# Effect of Nanofillers on Tribological Properties of Polymer Nanocomposites: A Review on Recent Development

**DOI:** 10.3390/polym13172867

**Published:** 2021-08-26

**Authors:** Jia Xin Chan, Joon Fatt Wong, Michal Petrů, Azman Hassan, Umar Nirmal, Norhayani Othman, Rushdan Ahmad Ilyas

**Affiliations:** 1 School of Chemical and Energy Engineering, Faculty of Engineering, Universiti Teknologi Malaysia, Johor Bahru 81310, Malaysia; jxchan2@live.utm.my (J.X.C.); jfwong4@live.utm.my (J.F.W.); norhayani@utm.my (N.O.); ahmadilyas@utm.my (R.A.I.); 2 Faculty of Mechanical Engineering, Technical University of Liberec, Studentská 2, 461 17 Liberec, Czech Republic; michal.petru@tul.cz; 3 Center of Advanced Mechanical and Green Technology, Faculty of Engineering and Technology, Multimedia University, Jalan Ayer Keroh Lama, Melaka 75450, Malaysia; nirmal@mmu.edu.my

**Keywords:** friction, wear, tribology, nanocomposites, carbon-based nanofillers, metal oxide nanofillers, silicon-based nanofillers

## Abstract

Polymer nanocomposites with enhanced performances are becoming a trend in the current research field, overcoming the limitations of bulk polymer and meeting the demands of market and society in tribological applications. Polytetrafluoroethylene, poly(ether ether ketone) and ultrahigh molecular weight polyethylene are the most popular polymers in recent research on tribology. Current work comprehensively reviews recent advancements of polymer nanocomposites in tribology. The influence of different types of nanofiller, such as carbon-based nanofiller, silicon-based nanofiller, metal oxide nanofiller and hybrid nanofiller, on the tribological performance of thermoplastic and thermoset nanocomposites is discussed. Since the tribological properties of polymer nanocomposites are not intrinsic but are dependent on sliding conditions, direct comparison between different types of nanofiller or the same nanofiller of different morphologies and structures is not feasible. Friction and wear rate are normalized to indicate relative improvement by different fillers. Emphasis is given to the effect of nanofiller content and surface modification of nanofillers on friction, wear resistance, wear mechanism and transfer film formation of its nanocomposites. Limitations from the previous works are addressed and future research on tribology of polymer nanocomposites is proposed.

## 1. Introduction

The science of tribology studies design, friction, wear and lubrication of interacting surfaces in relative motion [1]. Formerly, significant attention had been paid to metal with metal, and also metal with ceramic since metals and ceramics are conventional tribo-pair materials. In recent decades, polymeric materials are fast replacing these traditional materials in mechanical components due to their easy fabrication, lightweight, excellent chemical resistance, self-lubricating properties and uncalled-for maintenance. Although polymeric materials are popular in addressing tribology-related challenges in industries, their low mechanical properties, thermal conductivity and stability, and high thermal expansion avert their applications under high pressure and velocity (PV) operating conditions [2]. Conventional fillers such as metallic powdery filler, mineral fillers, carbon fibers (CF) or natural fibers are commonly used in improving their mechanical, thermal and tribological properties, especially at extreme operating conditions [3,4,5,6,7,8]. The perpetual pursuit of more advanced materials drives the development of polymer nanocomposites. Owing to their extensive surface area-to-volume ratio, nanofillers can significantly influence properties’ tuning at very low filler loading. Multifunctional nanofillers broaden the potential applications of polymer nanocomposites, allowing them to be tailored to a specific application, for instance, tribo-components for extreme operating temperature, working environment with unacceptable presence of lubricants and highly corrosive environment. These polymer nanocomposites do not only exhibit promising tribological behavior, some of them can also demonstrate good electrical conductivity for the usage in a micro-system or self-healing functionality for mechanical parts where maintenance works can hardly be conducted [4]. Newly developed polymer nanocomposites for tribological usage are the engineering solution for maximizing cost effectiveness by reducing material, wastage, and energy consumptions [9,10].

**Figure 1:**
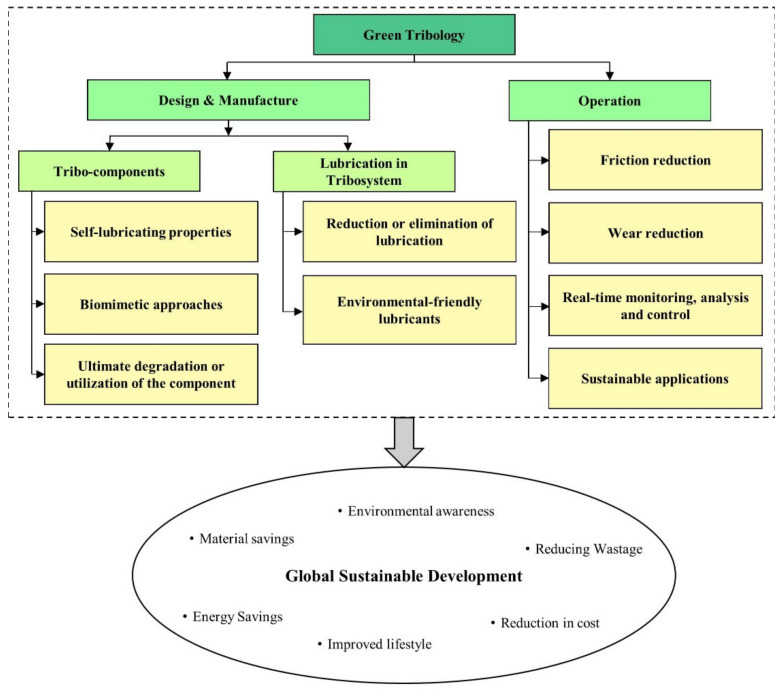
Green tribology and its important characteristics towards global sustainable development.

In today’s world, the term ‘green’ is widely used in almost all engineering practices. The term ‘green’ simply defines a specific method or process used that does not cause harm to the flora and fauna ecosystem [11]. Taking tribology for example, one can define ‘green tribology’ as the science and technology of tribological aspects related to the ecological balance of the environmental and biological impacts [12]. Nosonovsky and Bhushan formulated principles of green tribology from green chemistry [13]. The main objective is in the savings of energy and materials while maintaining a sustainable environment and a good quality of life. Figure 1 shows an infographic view on green tribology and its major components [13,14]. The figure summarizes crucial factors, namely defined in the ‘Design and Manufacturing’ and ‘Operations’ clusters, which are vital in the realization of an effective ‘global sustainable development’. To name but a few, energy savings, improved lifestyle, reduction in cost, environmental awareness, reducing wastage and material savings are the key elements towards an effective global sustainable development [15,16,17].

**Figure 2:**
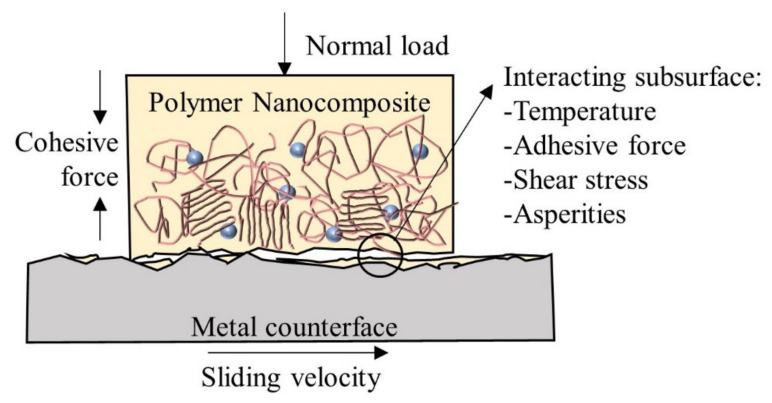
Parameters that have a strong influence on transfer film development.

Tribological properties of polymer are not intrinsic but are specific to the sliding system. Due to the viscoelastic properties of polymers, their tribological behavior is mainly dependent on the nature of the material and its counter-face, sliding surface roughness, contact pressure, velocity and temperature. These parameters determine the real contact area and the formation of transfer film, which contribute to different coefficient of friction (COF) and wear behavior [18,19,20]. Polymeric transfer film, that is usually developed during polymer-metal or polymer-polymer sliding, is the key factor in polymer tribology, as it will eventually change the contact surface. This is also an advantage of polymer-metal sliding over metal-metal sliding under a boundary or mixed lubrication regime, with the absence of lubricants. For polymer-metal tribo-pairs, material transfer always occurs from the polymer to metal counter-face. Figure 2 illustrates the crucial factors affecting the formation of thin transfer film onto the metal counter-face during a typical tribological wear test [21]. During the formation of film transfer, it is worth noting that the wear resistance of the “harder” material could be enhanced since the film transferred could act as a protecting element on the “harder” material [14]. Among the factors affecting the tribological behavior of polymer, inclusion of nanofiller significantly changes the nature and surface roughness of the material. It is worth noting that different sub-surfaces of material may experience different shear stress, temperature and adhesive force due to the heterogeneity of the polymer nanocomposite.

So far, the recent reviews of polymer nanocomposites have focused on the processing techniques, applications, and mechanical, thermal and other physical properties [22,23,24,25,26]. To the authors’ knowledge, there are only a few review papers found on tribological properties of polymer nanocomposites. Studies on tribological behavior of polymer nanocomposites up to the year 2017 were reviewed [27,28]. Later, review papers on tribological properties of polymer nanocomposites were limited to epoxy-, polyurethane-based nanocomposites coatings [29] and poly(ether ketone) nanocomposites [30]. However, to date, a comprehensive and up-to-date review for the tribological studies on thermoplastic and thermoset polymer nanocomposites conducted since 2017 has not been reported. Thus, this work will comprehensively review and discuss the latest scientific advances in polymer nanocomposites designed to enhance tribological properties under different operating conditions. The primary concern of the study is on how various nanofillers, and their compositions influence the friction and wear behavior of thermoplastic and thermoset polymers. The effect of surface modifications and sliding conditions on the tribological performance will be briefly reviewed. The versatility of transfer film formation to different nanocomposite systems and adhesive sliding conditions will also be examined. This article summarizes the challenges encountered and suggestions in advancements of polymer nanocomposites for tribological applications.

## 2. Tribological Performance of Polymer Nanocomposites

Polymers are modified by incorporating appropriate fillers to fulfil the requirements of a particular application and overcome their drawbacks. Nanotechnologies have benefited tribological research in terms of the distinguished characteristics in surface, volume and quantum dimensions of nanofillers [31,32,33]. Nanofillers are fillers having at least one dimension in the range of 1 to 100 nm, and they can be classified into various shapes depending on the number of nano-dimensions [22,34]. Nanofillers with one nano-dimension are known as nanoplates or nanosheets, those with two nano-dimensions are known as nanofibers and nanotubes, while those with three nano-dimensions are referred to as nanoparticles. Nanofillers have a principal function highly dependent on their structure [22,34]. Sheet-like nanofillers are normally composed of layers stacked with van der Waals gaps in between and exhibit excellent liquid and gas barrier properties. Attributing to their high aspect ratio, nanosheets, nanoplates, nanotubes and nanofibers generally have high load-carrying capability. Halloysite, as an open-ended nanotube, can allow some molecules to enter and perform special effects, such as polymer crystal nucleation, whereas nanoparticles of a proper concentration can achieve good balance in modulus, strength and ductility. In general, nanofillers enhance the tribological performance of polymers by increasing the load-bearing capacity, preventing sub-surface cracks, lubricating the sliding interface and increasing the thermal conductivity and thermal stability of the polymer. In this work, tribological studies are classified and reviewed according to filler types, in which the effects of incorporated fillers and their content are emphasized. The tribological performance of filled polymer nanocomposites under different testing conditions, such as applied load, sliding velocity, temperature, dry or lubricated condition, is also discussed.

### 2.1. Carbon-Based Nanofillers

Carbon-based materials have been known as one of the most important materials in nanotechnology. Their unique carbon structures have contributed to their diverse and superior physical properties, making them a popular subject of research in material science [35,36,37], particularly in tribology. Carbon nanomaterials can be classified into zero-dimensional (0D) represented by fullerenes, one-dimensional (1D) for carbon nanotubes (CNT) and carbon nanofibers (CNF), two-dimensional (2D) for graphene and graphene oxide (GO) and three-dimensional (3D) represented by nano-diamond (ND). The classifications of nanomaterials based on their dimensions are well-defined in [38].

CNT is the allotropes of carbon with sp^2^ hybridization, which is formed in a cylindrical structure. It is typically a cylinder of graphene, commonly used to improve the tribology performance of polymers. CNT is comprised of multi-walled CNT (MWCNT) and single-walled CNT (SWCNT). Due to the lubricating effect of MWCNT, the reinforced polytetrafluoroethylene (PTFE) nanocomposites experienced about 33% and 3% reductions of wear rate (WR) and COF, respectively [39]. This is also attributed to the good van der Waals interaction of MWCNT with PTFE, maximizing reinforcement effects of MWCNT, and subsequently increasing the load-carrying capacity [40]. Uniform distribution of MWCNT in high density polyethylene (HDPE) matrix also reduced wear loss by imparting resistance against plastic deformation during gear wear testing [41].

**Figure 3:**
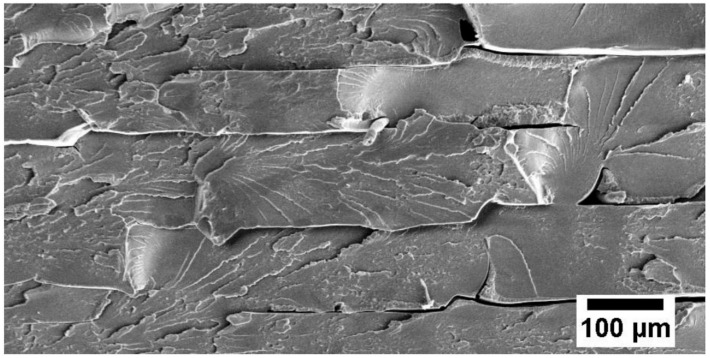
Scanning emission microscopic image on fracture surface of MWCNT/PEEK nanocomposites with 3 wt.% MWCNT loading (Reproduced with permission from M. F. Arif, H. Alhashmi, K. M. Varadarajan, J. H. Koo, A. J. Hart, S. Kumar, Composites Part B: Engineering; published by Elsevier, 2020) [42].

The wear resistivity of MWCNT/poly(ether ether ketone) (PEEK) showed different results with the incorporation of 1 wt.% MWCNT [42]. It reduced the COF of the nanocomposites from 0.25 to 0.08 but increased the WR with the reinforcement of MWCNT in PEEK nanocomposites. Lower hardness and higher multiscale porosity were observed for MWCNT/PEEK nanocomposites, as evident in Figure 3, which illustrates the visible interfaces between beads. This contrasting effect of MWCNT can be explained by previous works [43,44]. It is noteworthy that the frictional wear and COF were influenced by the nature of the pristine polymer matrix and structure of the nanofiller. The structure of the nanofiller is mainly characterized by the radius of annular formations of MWCNT, which is closely related to the degree of aggregation of the nanofiller and the level of interphase interactions between the matrix and nanofiller.

To enhance interfacial interactions with the matrix, the surface of MWCNT was functionalized with different functional groups [45,46,47,48]. Remanan et al. functionalized MWCNT with carboxylic group before incorporating it into polyaryletherketone (PAEK) [45]. The development of hydrogen bonding between MWCNT and PAEK led to hardness and wear resistance improvement. Polyimide (PI) reinforced by amino-functionalized MWCNT (CNTN) demonstrated a great reduction of COF and WR as compared to pure PI and carboxyl-functionalized nano-molybdenum disulfide (MoS_2_-MA)-reinforced PI [46]. The incorporation of CNTN converts the adhesive wear of pure PI into fatigue wear, displaying a flat wear surface with cracks caused by shear stress. Since CNTN offers a strong interfacial interaction with PI and hinders the desquamation of large wear debris, small fragments were observed. Similar wear debris was observed in [49]. In a recent study, MWCNT were functionalized through carboxylation, silanation, carbonylation and amination [47]. Among types of functionalization considered in this study are, silanized MWCNT/polyoxymethylene (POM) nanocomposite, which showed the best tribological performance, followed by those aminated, carbonylated, acid-treated and lastly the pure MWCNT. Unmodified MWCNT contributed to significant enhancement but better interfacial adhesion between modified MWCNT, and POM further improved reinforcement and prevented stress concentration [47,50]. It was highlighted that the strength, stiffness, and toughness of materials have a great effect on wear behavior.

Higher MWCNT content (up to 1.0 wt.%) led to lower WR and COF, attributing to the increasing heat dissipation effect [47,51]. Ascribed to the abrasive action of MWCNT aggregates, WR increased at contents beyond 1 wt.% MWCNT [47], whereas for thermosetting epoxy, MWCNT loading up to 3 wt.% significantly reduced WR due to the formation of stable transfer film [52]. The further increase of MWCNT loading increases the WR due to the agglomeration at the contact zone, resulting in non-protective dense debris. In contrast with the downward trend of COF observed in MWCNT/POM [47], increasing MWCNT in epoxy resin significantly increased surface roughness, which led to the upward trend of COF. Besides, MWCNT nanocomposites exhibited higher WR [47,50,51] but lower COF [47,50] at higher applied load. Higher applied load crushes the particles on the sliding surface into smaller sizes to reduce the abrasive effect, at the same time filling up the asperities, contributing to a larger real contact area. This promotes the formation of transfer film. Due to weak adhesion between MWCNT/epoxy debris and the asperities, the continuous flushing of particles from the interspace led to higher COF and lower WR at higher applied load, which is different compared to the result reported for POM and POM/PTFE nanocomposites [47,50].

Ultrahigh molecular weight polyethylene (UHMWPE) coating was incorporated with SWCNT and coated on titanium and its alloy surface, specifically for biomedical applications [53]. The configuration and the flow of testing conducted are similar to the work reported by Azam and Samad [54], where constant sliding speed at 0.1 m/s and varied normal loads were applied. Pristine UHMWPE coatings tested under normal loads of 7 and 9 N did not fail after 5000 cycles but failed after ~3600 cycles under 12 N normal load [53]. It is worth highlighting that this result is different from those reported by Azam and Samad, where the same UHMWPE coating, coated with the same technique, failed at ~5000 cycles under 9 N normal load [54,55]. The processing and grades of UHMWPE can hardly influence tribological performance [56]. Thus, the possible factor that resulted in the difference in results is the adhesiveness of the UHMWPE coating on different substrates. Evenly distributed SWCNT with at least 1.5 wt.% content was found efficient in anchoring the polymer chains to reduce the material pull-out at 12 and 15 N. Higher contents led to agglomeration and uneven morphology of coatings. All coatings underwent a combination of adhesive and abrasive wear.

The effects of SWCNT and MWCNT on the tribological performance of vinyl ester nanocomposites were compared [57]. MWCNT reduced the COF of the nanocomposites, but incorporation of SWCNT at high sliding speed increased the COF. The study suggested that the presence of MWCNT in the debris contributed to the lubricating effect due to its strong interactions with the polymer matrix, while SWCNT in the debris acted as a third body during the sliding motion. However, SWCNT/vinyl ester nanocomposites have lower WR compared to MWCNT nanocomposites. In terms of friction stability, the 0.15 wt.% SWCNT nanocomposite exhibited the best result, followed by neat polymer and 0.15 wt.% MWCNT nanocomposites. Both unexpected results on WR and friction stability might be due to the rolling contact of debris on the sliding contact.

Several studies did not mention the type of CNT used in their research, and thus the discussion on these studies will generalize the nanofiller as CNT, which were supplied from the same company. The incorporation of CNT into poly(phthalazinone ether sulfone ketone) (PPESK) film [58] and acid-treated CNT into epoxy resin [59] reduced the COF and WR of their respective nanocomposites. This is ascribed to the high bearing capacity of CNT and its protection to the matrix surface from severe plowing damage, promoting the formation of small and thin debris. CNT has a better wear-reducing effect than graphitic carbon nitride nanosheets (g-C_3_N_4_) [58] and molybdenum disulfide (MoS_2_) [59], but not in the case of COF reduction. The structure of CNT provided more reinforcing effect, while the lamellar structure of g-C_3_N_4_ and MoS_2_ is easier to be sheared off. CNT- and acid-treated CNT-filled epoxy nanocomposite coatings were compared [60]. Both types of CNT reduced COF as well as the wear width and depth. In agreement with other research on acid-MWCNT, acid-CNT presented better properties than CNT as the carboxyl groups on its surface contributed to better dispersion. The worn surface of acid-CNT/epoxy was denser and more compact.

CNT and CNF are symbolic 1D nanofillers. The primary difference between them is their morphology. CNF has a structure where graphene planes are arranged as stacked cones from the fiber axis, exposing the edge planes on the interior and exterior surfaces of the fiber. Incorporation of 0.5 wt.% of CNF into UHMWPE/block copolymer of polypropylene with linear low-density polyethylene (PP-b-LLDPE) blend improved the wear resistance by 5 times and reduced the COF by half [61]. Compared to aluminum hydroxide oxide and copper (Cu) nanoparticles, CNF exhibited the lowest surface roughness and thus led to the highest wear resistance. CNF exhibited similar anti-wear and friction-reducing performance as CNT when compared to MoS_2_ [62].

Besides, it was found that coiled CNF afforded thermoplastic polyurethane (TPU) better wear resistance as compared to the one reinforced with straight nanofibers due to its better reinforcement effect [63]. Hollow CNF (HCNF), a carbon analogous to MWCNT with a thicker tube wall, was used to enhance the tribological performance of PI [64]. Results were obtained under different sliding conditions, where dry, water-lubricated and paraffin oil-lubricated sliding showed decreasing COF and WR with increasing HCNF content, while under dry sliding, COF values fluctuated [64]. Lubrication of oil offered the best tribological performance, whereas water lubrication significantly promoted wear of the nanocomposites. This is because water molecules diffusing into the PI network caused swelling of the matrix and removal of materials.

The superior mechanical property and high thermal conductivity of graphene led to its excellent tribology applications in polymer nanocomposites [65]. The atomically smooth surface and weak van der Waals force between the graphene layers ease the interlayer sliding and contribute to its self-lubrication characteristic [66]. Furthermore, the 2D structure of graphene with high specific surface area is also good for load transferring. Aliyu and co-workers conducted a series of research on tribological properties of graphene-reinforced UHMWPE nanocomposite in bulk and coating form for mechanical bearing applications. The effect of graphene loading, normal load and sliding speed on the friction and wear behavior of bulk graphene/UHMWPE nanocomposites [67] and coating [68] were investigated. Different from the expected lubricating effect, graphene exhibited an anchoring effect in bulk UHMWPE nanocomposite to prevent polymer chains from sliding over one another. Thus, COF increased with the increasing graphene loading (0.1–0.5 wt.%) [67]. However, the load transfer ability of graphene contributed to improved wear resistance with an optimum content of 0.25 wt.%. Nanocomposite coating exhibited a similar trend as the bulk sample for WR, but the addition of graphene and its content resulted in a negligible change in COF [68]. Both bulk nanocomposite and coating experienced purely abrasive wear.

Graphene/UHMWPE nanocomposites were then tested under different conditions. Subjected to the surface softening, increasing PV caused a progressive decrease in COF, but an increase in WR, regardless of whether the sample was in bulk or coating form. Graphene/UHMWPE nanocomposite coating failed at 8 MPa, 0.1 m/s, while wear track surface morphology of the bulk sample changed from a smooth surface to a surface with ridges or protrusion when speed increased from 0.1 to 0.5 m/s. A drastic increment of WR of 757% and a very rough wear surface under 0.75 m/s indicated a significant change in the wear mechanism and that the sliding speed is over the velocity limit of the nanocomposites. The overall PV limit of 0.25 wt.% graphene/UHMWPE bulk nanocomposite and 1 wt.% graphene/UHMWPE nanocomposite coating was ascertained to be 6 MPa·m/s and 4 MPa·m/s respectively, which indicate better performance than some of the commercially available materials.

In order to simulate the contact conditions in thrust bearing, a 1 wt.% graphene/UHMWPE nanocomposite coating was tested under dry and base oil-lubricated conditions using ring-on-disc contact configuration [69]. Similar to the previous study conducted using pin-on-disc configuration, elevated PV reduced COF but promoted wear under dry sliding. The lubricating effect of base oil significantly enhances the tribological performance of nanocomposites. Before the contact pressure reached 3.1 MPa, where the lubricant was squeezed out of the contact, elevated contact pressure showed no significant effect on the COF but caused an increase in WR. In the presence of lubricant, WR of nanocomposites reduced and was subsequently increased when the sliding speed increased from 1, 1.5 to 2 m/s. The initial reduction of WR is due to the predominant peeling effect over the adhesive wear mechanism.

A positive impact of graphene on COF and more specifically in WR of PEEK nanocomposites was observed under the water-lubricated sliding condition [70]. A combination of abrasion and adhesion wear predominated in neat PEEK and 1 wt.% graphene/PEEK nanocomposites, while 10 wt.% graphene contributed to fatigue wear. Grooves and unevenly eroded patches were exhibited on neat PEEK, while scratches and plastic fragments were found on the surface of 3 wt.% graphene nanocomposites. The scratches and grooves progressively disappeared with increasing graphene content, leaving a smooth and homogenous surface with some subsurface cracks. The evolution of the wear mechanism is due to the variation of hardness and toughness of the composites with graphene content. However, the work by Arif et al. showed that 3 wt.% graphene/PEEK nanocomposites had lower COF but higher WR due to their lower hardness and increased porosity when compared to neat PEEK samples [42]. The different effect of graphene on PEEK is probably due to the different fabrication methods and presence of lubricant in both studies.

**Figure 4:**
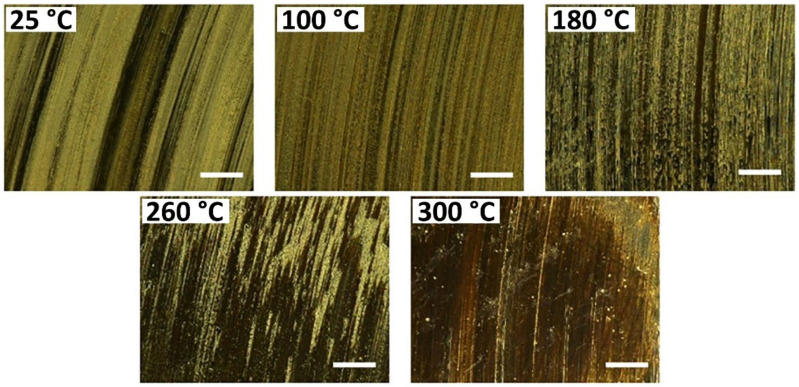
Optical microscopic images of steel pins after sliding at different temperatures against graphene/ATSP nanocomposite coatings (Reproduced with permission from K. Bashandeh, P. Lan, J. L. Meyer, Tribology Letters; published by Springer Nature, 2019) [66].

Poly(2-butylaniline)/epoxy [71] and aromatic thermosetting copolyester (ATSP) nanocomposite coating [66] showed improved tribological properties and wear life respectively, after incorporation of graphene. Graphene promotes the formation of thin transfer film during the sliding of thermoset nanocomposites, by increasing the adhesive force at the interface [66,72,73]. Enhanced thermal conductivity and stability of the coating are also responsible for the tribological properties’ improvement [66,71,73]. Well-dispersed graphene within the polymer matrix formed a thermally conductive network to prevent heat accumulation on the contact surface that may promote excessive removal of material. Graphene/ATSP coatings demonstrated a great potential in tribological applications over a wide range of temperatures up to 300 °C [66]. COF and WR of the nanocomposite coatings continuously decrease at elevated temperatures, reaching 53% and 69% reductions at 300 °C due to the formation of continuous transfer film, as shown in Figure 4.

**Figure 5:**
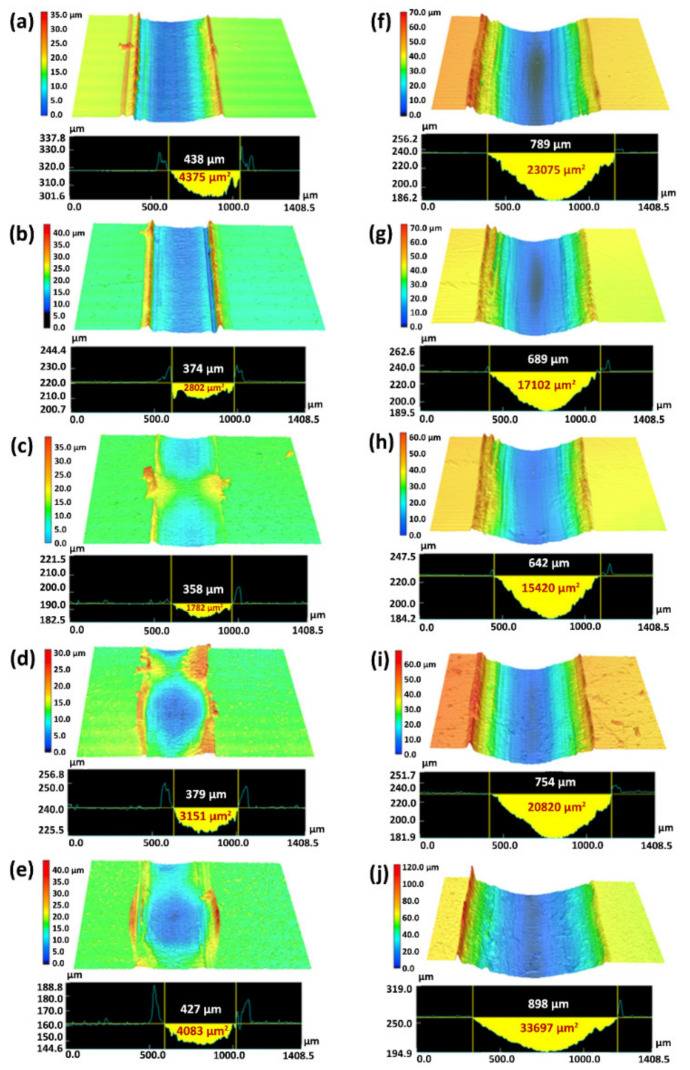
The 3D morphologies and profile of wear track: (**a**) pure PI, (**b**) 0.25 FG/PI, (**c**) 0.5 FG/PI, (**d**) 1.0 FG/PI, (**e**) 2.0 FG/PI under dry friction condition and (**f**) pure PI, (**g**) 0.25 FG/PI, (**h**) 0.5 FG/PI, (**i**) 1.0 FG/PI, (**j**) 2.0 FG/PI under seawater lubrication condition (Reproduced with permission from S. Zhou, W. Li, W. Zhao, C. Liu, Z. Fang, X. Gao, Colloids and Surfaces A; published by Elsevier, 2019) [75].

Unlike other research works which incorporated unmodified graphene, evenly dispersed sulfonated graphene in polyurethane [74] and chloroform-modified fluorinated graphene (FG) in PI [75] were documented. COF of the FG/PI nanocomposites were lower than that of pure PI, regardless of the friction conditions. The trend of COF and WR as a function of FG content initially decreased, reaching the minimum at 0.5% FG, before increasing. A similar trend was observed in the silane-modified graphene/epoxy nanocomposite, where 0.3 wt.% loading exhibited minimum values [73]. Modified graphene exhibited enhanced interfacial adhesion with the polymer matrix. COF of FG/PI nanocomposites under seawater lubrication were 40.2% lower than dry condition as water film reduced the contact area between frictional pairs. However, the worn surface morphologies of the samples, illustrated in Figure 5, revealed that PI nanocomposites were not suitable for sliding under seawater environments. Samples experienced extensive damage under the environment, ascribing to the hydroscopicity nature of PI previously discussed under HCNF/PI nanocomposites [64]. Furthermore, dopamine-coating nanographite facilitates the formation of transfer films to effectively reduce the adhesion and fatigue wear and improve 52% of the wear resistance of epoxy nanocomposites [76].

GO is graphene with various oxygen-containing functionalities, such as epoxide, carbonyl, carboxyl and hydroxyl groups [77]. Owing to the self-lubricating and reinforcing effect of GO, GO-reinforced polypropylene (PP) [78] and PI [79] nanocomposites possess enhanced tribological properties. Reinforcing ability inhibited microcracking of the specimens and promoted transfer film formation. Its lubricating effect reduces the shear force of nanocomposites [39]. Other than that, incorporation of GO also induced higher crystallinity in the polyamide 6 (PA6) nanocomposite [80]. High crystallinity is proven to contribute positively to tribological performance. Concentration of GO has to be sufficiently high (2 wt.%) to show a significant reduction in the wear of GO/UHMWPE nanocomposites under hip kinematic conditions [81]. Similar to MWCNT, incorporation of only 2 wt.% GO into the PTFE matrix was able to reduce the WR by about 36% [39], whereas in thermoset epoxy resin, GO provided a greater positive effect to tribological properties than CNT [59]. Compared to CNT, GO has a lamellar structure for better shear off, and also better interfacial interactions with epoxy through its functional groups, contributing to better stress transfer.

**Figure 6:**
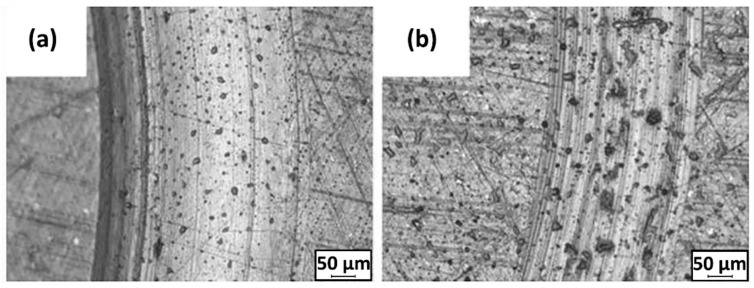
Optical photographs of worn surfaces of PI nanocomposites with 0.5 wt.% GO under (**a**) dry sliding and (**b**) seawater lubrication (Reproduced with permission from C. Min, C. Shen, M. Zeng, P. Nie, H. J. Song, S. Li, Monatshefte für Chemie—Chemical Monthly; published by Springer, 2017) [82].

In order to enhance the interaction between PP and GO, and promote homogenous dispersion, PP-grafted maleic anhydride (PP-g-MA) was added into the nanocomposite [78]. When GO content increased from 0.05 to 0.15 wt.%, COF and WR decreased regardless of the presence of a compatibilizer. Similar to the report regarding graphene/UHMWPE nanocomposites, PP and its nanocomposites exhibited increasing COF and WR with increasing load and sliding speed. This phenomenon often occurs in the dry sliding condition [82,83], whilst under the seawater lubricating condition, the dissipation of friction-induced heat is facilitated [82]. Moreover, a tribo-chemical reaction can occur between the GO/thermoplastic PI nanocomposite, metal counter-face and Ca^2+^, Mg^2+^ ions in the seawater. As a result, calcium carbonate (CaCO_3_) and magnesium hydroxide (Mg(OH)_2_) layers, which possess self-lubricating properties, are deposited on the sliding surface. GO/PI nanocomposites exhibited lower COF and WR under seawater lubrication than dry sliding. Comparing the optical photographs of worn surfaces under dry sliding and seawater lubrication, the corrosive effect of seawater can be clearly observed, as depicted in Figure 6.

Furthermore, GO can be surface-treated to yield better interface properties and tribology performance as compared to untreated GO [84,85,86]. Amino groups [85], polyetheramine-functionalized GO [86] and remaining oxygenic groups of GO interact well with the epoxy system to resist crack propagation and fatigue wear. Whereas plyhedral oligomeric silses-quioxane (POSS)-functionalized GO can react with PI through amidation and self-fix in the resin through its “plate-anchor” structure [79]. In these works, an extremely smooth, thin, strong and continuous transfer film was formed on the counter-face. This film is promoted by small wear debris. The transfer film in turn reduces the shear stress strength of the nanocomposite surface.

COF and WR of epoxy coating [87] and bismaleimide (BMI) resin [88] showed obvious reductions after the addition of reduced GO (RGO). The fatigue deformations of pure epoxy coating reduced, and fewer deep furrows were observed, indicating a weaker shear force between the friction pair [87]. Tribological properties of the RGO/BMI nanocomposites were compared to those of zirconium dioxide (ZrO_2_)/BMI and MoS_2_/BMI [88]. The results indicated that RGO/BMI nanocomposites had better anti-friction properties than ZrO_2_/BMI but poorer than MoS_2_/BMI. On the other hand, RGO/BMI nanocomposites revealed almost similar volume of WR to that of MoS_2_, but higher than ZrO_2_/BMI.

Similar to GO, surface-treated RGO promotes the formation of uniform, continuous and thin self-lubricating transfer film. The transfer film is responsible for the COF and WR reduction [76,89,90]. Liu et al. incorporated 0.6 wt.% of polytriazine (PTZ)-treated RGO into BMI resin and found that the dispersibility of filler in the matrix was improved [89]. PTZ-treated RGO/BMI nanocomposites showed 10% and 20% lower WR and COF respectively, as compared to RGO/BMI nanocomposites. The load transfer between the polymer and RGO is easier due to enhanced interfacial interaction and compatibility of PTZ-treated RGO. Another recent study modified RGO with dehydrated ethylenediamine (EDA-RGO) to enable covalent bond formation between the epoxy matrix and nanofiller [90].

ND represented 3D carbon-based nanofillers were incorporated into UHMWPE [91]. Similar to other carbon-based nanofillers, a low amount of ND is able to improve the tribological performance of the polymer matrix. This is attributed to the self-lubrication effect of ND that reduced the adhesion, shear stresses and ploughing phenomena between the tribo-pair, since ND act as anemometric ball bearings. The high thermal conductivity of ND also facilitated the dissipation of frictional heat during the wear process. However, surface modification of ND with methytriethoxy silane (MTS) is required to overcome the agglomeration of ND.

### 2.2. Silicon-Based Nanofillers

Clay-reinforced polymer nanocomposites are prospective due to their low cost. However, the extent of penetration of polymer chains into galleries of clays is limited. Thus, laminated silicates are normally modified by surface-active substances [2]. High-strength, stiffness and hardness epoxy that resulted from the incorporation of 3-aminopropyltrimethoxysilane-modified montmorillonite (MMT) [72] and trimethyl stearyl ammonium-modified nanoclay [92] showed better tribological properties. The analysis of variance (ANOVA) indicated that filler content, applied load, sliding speed and duration have significant effects on the wear performance of nanoclay/epoxy nanocomposites [92]. Filler content and applied load had the greatest influence on the wear performance of nanoclay/epoxy nanocomposites. Increasing nanoclay content up to 4 wt.% enhanced the reinforcement to epoxy resin [92] and features of transfer film to protect the sliding surface [54,72,92,93,94]. Similarly, optimum tribological properties were observed at 4 wt.% of Cloisite 30B nanoclay/polyvinylidene fluoride (PVDF) nanocomposites [95]. Nevertheless, wear increased at higher sliding speed, time and load, as the localized temperature at the contact region is higher under these conditions [54,92].

**Figure 7:**
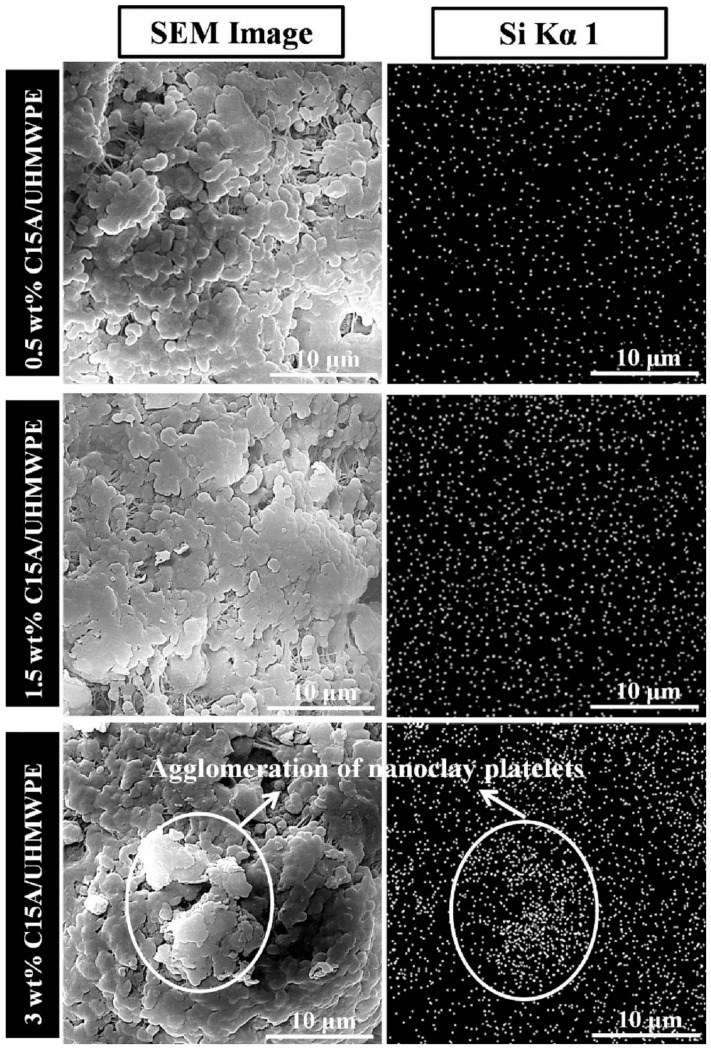
Scanning electron microscopic images of C15A/UHMWPE nanocomposites with 0.5, 1.5 and 3 wt.% loading, along with energy-dispersive X-ray spectroscopic elemental maps for silicon (Reproduced with permission from M. U. Azam, M. A. Samad, Progress in Organic Coatings; published by Elsevier, 2018) [54].

In another study, UHMWPE nanocomposites were reinforced by different organoclays, namely quaternary dimethyl dehydrogenated ammonium-modified Cloisite 15A (C15A) nanoclay [93,94], primary octadecyl ammonium ion-modified I30E [93] and quaternary octadecyl ammonium ion-modified I28E nanoclays [93]. All nanocomposites exhibited lower WR and COF than that of pristine UHMWPE. Metal oxides present in the clay enhanced the adhesion between transfer film and the counter-face by forming covalent bonds. Among the nanoclays, modified C15A/UHMWPE nanocomposites exhibited the best tribological performance due to their exfoliated structure. C15A anchored polymer chains to improve load-bearing capacity and resistance on plastic deformation [54,94]. COF [94] and WR [54,94] initially decreased with the increase in unmodified and modified C15A content, then increased due to agglomeration, as shown in Figure 7. COF of coatings showed no appreciable changes with different C15A loadings [54].

Halloysite nanotube (HNT) is an aluminosilicate clay nanofiller with nanotubular structure, high meso/macroscopic pore structure and large specific surface area. It has advantages over CNT in terms of high stability, ease of disposal and reusability [96]. Polyamide 11 (PA11) incorporated with 3 wt.% of HNT experienced reductions of about 38% and 13% in WR and COF respectively, attributing to the formation of transfer film [97]. Different results were reported in HNT/PTFE nanocomposites [96]. The wear resistance properties improved but COF increased. This is attributed to the same order of magnitude of HNT with the dimensions of PTFE single crystals, which allows them to intertwine together and impede the drawing out of PTFE crystals during sliding. An obvious increment of COF can be observed only above 2 wt.% of HNT/PTFE nanocomposites.

The surface was then modified by poly(methyl methacrylate) (HNT-PMMA), sodium dodecyl sulfate (HNT-SDS) and carboxylic acid (HNT-COOH) to improve its dispersibility in PTFE [98]. The wear mechanism turned abrasive from adhesive wear after the modification of HNT. COF of modified HNT/PTFE nanocomposites varied considerably with sliding time and were significantly greater than of pristine HNT/PTFE. These results indicate that the addition of modified HNT led to structure modification in the PTFE matrix and increased the surface roughness, which was not observed in pristine HNT/PTFE. It also significantly reduced the WR, which then retards the formation of transfer film. Moreover, other researchers reported that possible surface modifications on HNT are vinyltrimethoxy silane (VTMS) and N,N’-ethylenebis(stearamide) (EBS). VTMS-treated HNT improved the wear resistance of the HDPE matrix [84], whereas EBS formed hydrogen bonds with HNT to improve the interfacial compatibility and lower COF [99].

In a recent study, UHMWPE was incorporated with wollastonite (calcium silicate) to improve wear resistance [100]. UHMWPE nanocomposites achieved at most six times lower WR at 1 wt.% wollastonite content. Throughout the sliding, UHMWPE nanocomposites were restructured, protruding wollastonite on the surface to form a more wear-resistant surface. At low wollastonite loading, transfer film of higher elastic modulus is promoted. This work highlights that the agglomeration of wollastonite at high content changed the supramolecular structure of UHMWPE from lamellar to spherulitic, where the structure becomes loose and disordered at higher wollastonite content. These large particles can easily detach from the specimen and act as abrasive particles on the friction path. IR spectroscopy evinced that wollastonite of higher content promoted the tribo-oxidation process.

**Figure 8:**
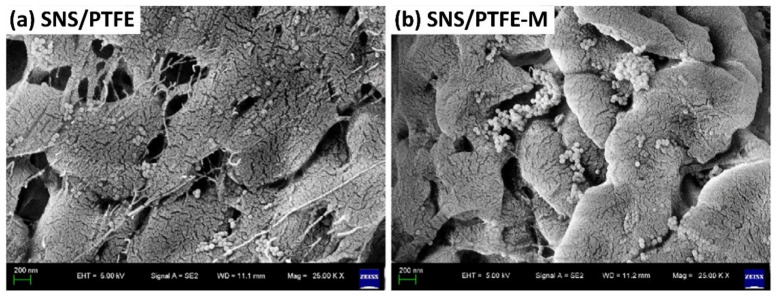
Scanning electron microscopic images of PTFE nanocomposites powder prepared by (**a**) in situ filling (2SNS/PTFE) and (**b**) the mechanical mixing method (2SNS/PTFE-M) (Reproduced with permission from G. Shi, Q. Wang, T. Sun, X. Yan, Journal of Applied Polymer Science; published by Wiley, 2020) [102].

Silicon dioxide (SiO_2_), also known as silica, is a common filler used for polymer materials for properties’ enhancement [101]. SiO_2_ nanospheres (SNS) were incorporated into the PTFE matrix as a solid lubricant [102]. In situ filling afforded an excellent dispersion in the PTFE matrix and increased the cohesive energy density of PTFE when compared to the mechanical mixing method, as shown in Figure 8. This result agrees with a similar finding on SNS/UHMWPE nanocomposites [103]. The incorporation of SNS into the UHMWPE matrix via in situ filling improved interface adhesion between the matrix and filler when compared to mechanical mixing, thus optimizing the functional properties of SNS, particularly in improving tribological performance.

**Figure 9:**
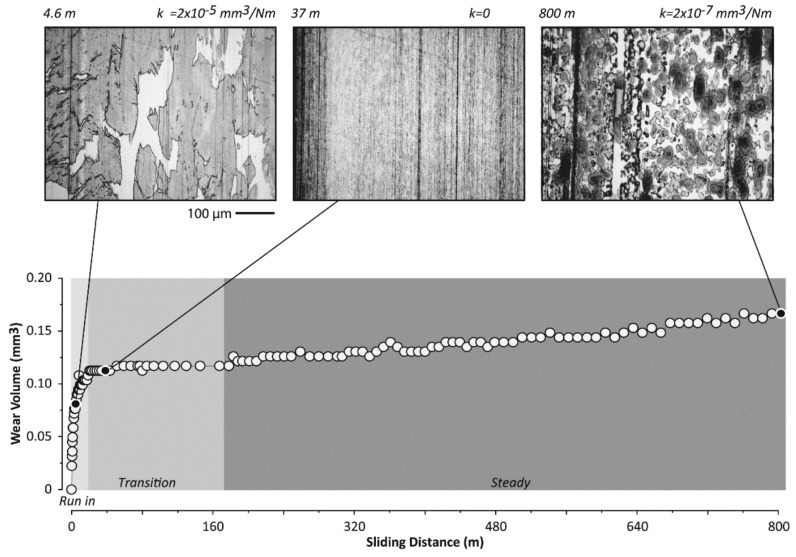
Micrographs of the transfer film morphology for run-in, transition and steady-state phases, corresponding to the graph of wear volume against sliding distance (Reproduced with permission from J. Ye, H. S. Khare, D. L. Burris, Wear; published by Elsevier, 2013) [106].

**Figure 10:**
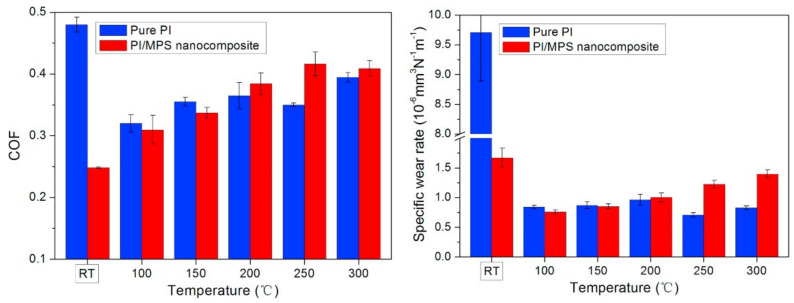
Friction coefficient and wear rate of PI and MPS/PI nanocomposites as a function of sliding temperature (Reproduced with permission from J. Ma, X. Qi, Y. Zhao, Q. Zhang, Y. Yang, Wear; published by Elsevier, 2017) [104].

Thermoplastic PI incorporated with mesoporous silica (MPS) was tested under different operating temperatures [104]. At room temperature, the WR and COF reduced after PI was filled with MPS. MPS/PI nanocomposites underwent tribo-chemistry evolution during sliding [105]. It decomposed and reacted with air to form a high-cohesion transfer film with great adhesion on the counter-face. The reduction of wear debris size due to MPS incorporation also promotes the formation of transfer film [105,106]. Transfer film morphologies evolved with sliding distance, as illustrated in Figure 9. In the run-in period, polymer debris were generated, deformed, removed, and replenished. Thin-region seeds which remain well-adhered replaced loose debris in the run-in period. The seeds grow and nucleate the transfer film over time and become small islands, which then merge to form a continuous transfer film. Nonetheless, steady-state wear does not exist in some cases, especially when adhesion between the transfer film and counter-face is weak or the abrasive wear mechanism is dominant. COF of MPS/PI nanocomposites increased linearly with the increase in temperature until 250 °C, then a slight decrement was observed at 300 °C, as shown in Figure 10. WR increased linearly from 100 to 300 °C, with all values being lower than the value at room temperature. Comparatively, MPS/PI nanocomposites demonstrated inferior tribological performance at elevated temperatures compared to pure PI, due to the weak interfacial strength between MPS and the matrix.

As epoxy is commonly used for anti-cavitation painting or coatings, its nanocomposite samples were prepared in bulk and as coating to be studied [107]. The effect of sliding distance towards WR was studied. WR of neat epoxy increased with the increase in sliding distance, presenting a maximum at 1000 m, before being stabilized. High stress between sliding surfaces at the initial transition period promotes wear until a steady state is achieved. A similar behavior was observed for 5% SiO_2_ nanocomposites with higher WR value, reaching a maximum at 2000 m, but they did not stabilize over the studied period. The wear resistance of 3% SiO_2_ nanocomposites was comparable to that of neat epoxy, with a minimum observed at 1000 m, and increasing wear with sliding distance due to the plasticizing effect of nano-silica. The neat epoxy coating showed comparable WR as its bulk polymer, but the 3% SiO_2_ nanocomposite coating exhibited a significantly higher value when compared to its bulk polymer nanocomposites. This is also attributed to the plasticizing effect.

**Figure 11:**
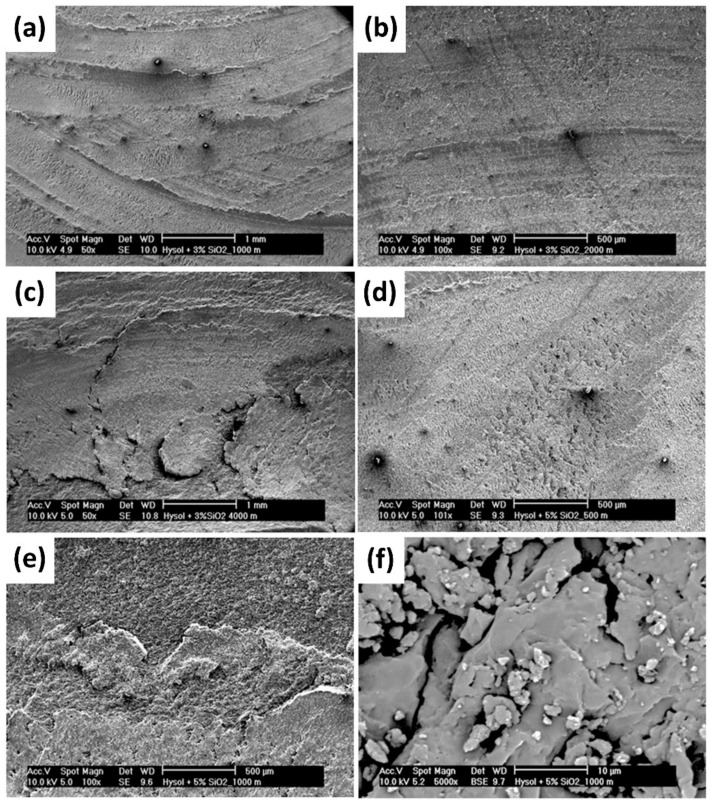
Wear tracks micrographs of 3% SiO_2_ nanocomposite at (**a**) 1000 m, (**b**) 2000 m and (**c**) 4000 m, and 5% SiO_2_ nanocomposite at (**d**) 500 m, (**e**) 1000 m and (**f**) detailed 1000 m (Reproduced with permission from J. Abenojar, J. Tutor, Y. Ballesteros, J. C. del Real, M. A. Martínez, Composites Part B; published by Elsevier, 2017) [107].

The wear mechanism changes of epoxy and its nanocomposites were also investigated [107]. For all sliding distances (500–4000 m), the epoxy showed abrasive and adhesive wear. A lubrication effect was observed in the micrograph of 3% SiO_2_ nanocomposites at 1000 m (Figure 11a), while adhesive wear and material removal were observed at 4000 m. The lubrication effect also lowered the COF of nanocomposites at 1000 m. However, 5% SiO_2_ nanocomposites showed more abrasive wear with fatigue cracks (Figure 11e,f), where many particles were found on the wear track. These particles may act as a third body, thus causing higher WR and COF. Fatigue cracks appeared in both nanocomposites, starting from a 2000 m sliding distance (Figure 11b,c), and contributed to higher COF. Thus, COF was found to increase with sliding distance.

### 2.3. Metal Oxide Nanofillers

Aluminum oxide (Al_2_O_3_), also known as alumina nanoparticles, has high strength, thermal conductivity and wear resistance. Owing to these properties, it is used in several studies to improve the wear resistance of polymeric materials [108,109]. However, it is worth mentioning that no clear relation has been observed between compressive/shear modulus and WR in alumina/PTFE nanocomposites [65]. Thus, types of filler and their promotion in transfer film formation are the dominating factors affecting WR of alumina/PTFE nanocomposites. Al_2_O_3_ nanoparticles have a friction-reducing effect by generating a transfer film with greater bearing capability [109]. However, both gamma phase Al_2_O_3_ (𝛾-Al_2_O_3_) and fumed Al_2_O_3_ nanopowder failed to act as an effective solid lubricant in the HDPE matrix [83]. This agrees with the work of Llorente et al., which mentioned that bare 𝛾-Al_2_O_3_ forms big aggregates in polysulfone (PSU) and acts as a highly abrasive third-body material [110]. Its incorporation and content increased the COF value. This is attributed to the better interaction between Al_2_O_3_ and epoxy than that with non-polar HDPE. After 𝛾-Al_2_O_3_ nanopowder was grafted with PSU chains, COF and WR of the filled PSU nanocomposites reduced by up to at least 12% [110]. Aggregate sizes reduced after 𝛾-Al_2_O_3_ had a better interface interaction with the PSU matrix. Grafting of short PSU chains with a lower degree of entanglement exhibited a better effect than long PSU chains.

The effects of alumina content and testing condition on tribological properties were documented [111]. Lower COF and WR values of the Al_2_O_3_/poly(methyl methacrylate) (PMMA) nanocomposites were observed at higher alumina concentration, but higher values were reported at higher applied load. The effect of alumina content on tribological properties of ortho cresol novalac epoxy (OCNE) nanocomposites [112] is in agreement with those of 𝛾-Al_2_O_3_/PSU, but in contrast with Al_2_O_3_/PMMA and Al_2_O_3_/PTFE nanocomposites [65]. COF of the Al_2_O_3_/OCNE nanocomposites increased with the increase in filler contents and sliding velocity but reduced when the applied load and sliding distance increased [112]. Furthermore, WR increased as the filler content, applied load and sliding distance increased, but decreased as the sliding velocity increased.

Incorporation of zinc oxide (ZnO) nanoparticles into OCNE resin has similar tribology responses as Al_2_O_3_/OCNE nanocomposites [112]. The increasing COF and WR values with the increasing ZnO content were also reported in polyamide (PA) nanocomposites [113], while UHMWPE nanocomposites reported an increasing trend in WR only [114]. This is attributed to its agglomeration, which then activates fracture at the interface point, roughness and three-body wear. Although the tribological performance deteriorated with increasing nanofiller loadings, introduction of ZnO nanoparticles significantly reduced WR at 5 wt.% ZnO/UHMWPE nanocomposite, while it reduced COF and WR of neat PA at the lowest content of 1 wt.% ZnO.

Work on lanthanum oxide (La_2_O_3_)/epoxy/PVDF nanocomposites demonstrated that La_2_O_3_ nanoparticles yielded better tribological performance in general as compared to nanocomposites with MoS_2_ nanoparticles [115]. This is attributed to the effectiveness of La_2_O_3_ nanoparticles in enhancing the thermostability and surface hardness, which limit the adhesive wear of nanocomposites with the counter-face. The continuity of the transfer film formed is higher in the case of La_2_O_3_-based nanocomposites. Due to the high surface activity of La_2_O_3_ nanoparticles, it is able to disperse well in the matrix of the epoxy/PVDF blend with the formation of an integrated fluoride network within the nanocomposites. Better wear resistance enhancement can be observed in the presence of lubricating oil as it can easily remain on the contact zone of the tribo-pair.

Cu and cupric oxide (CuO) are well-known as soft fillers, where they can effectively devour shear force applied on transfer film to reduce wear [116]. The incorporation of CuO [33] and Cu coated with silicon (Cu/Si) nanoparticles [117] reduced COF and wear scar width of the nanocomposites when compared to pristine UHMWPE and PA6, respectively. CuO acted as rigid abrasive particles to reduce the contact area, as well as enhancing the molecular entropy of the system. It also served as rigid stress receptors in the matrix to resist surface deformation. With this, the wear mechanism transformed from adhesive to fatigue wear. Metal Cu nanomaterials coated with inorganic materials (Si) exhibited properties similar to their parent nano-metal, with less potential of aggregation and oxidation.

### 2.4. Miscellaneous Nanofillers

Boron nitride (BN) is a hard ceramic material with excellent thermal stability and lubrication properties. It is often known as white graphite due to its planar hexagonal structure. Tribological properties of BN-based polymer nanocomposites are often evaluated under water or seawater lubrication, in comparison with dry sliding. BN has different crystalline structures, particularly cubic, hexagonal, amorphous and wurtzite lattices [118]. Under dry sliding, hexagonal BN (*h*-BN) nanoparticles reduced WR of PAEK nanocomposites by 22 times but contributed to higher COF [119]. This is attributed to the good interface interaction between *h*-BN and PAEK, which increases microhardness and lateral force on the wear track. Whereas under water-lubricated sliding, wear resistance of POM nanocomposites was enhanced by one order of magnitude after the addition of *h*-BN nanoparticles, and the COF value significantly reduced, especially at higher applied load [120]. The performance of *h*-BN/POM nanocomposites was less affected by applied load variation than neat POM. High applied load promotes the formation of continuous transfer film to fill up the roughness grooves and plateau areas on the counter-face. The presence of boric acid (H_3_BO_3_), boron trioxide (B_2_O_3_), BN, iron oxide and POM in the transfer film is observed in the gradient structure. These products of tribo-chemical reaction of *h*-BN (hydration) aligned at the outmost layer of the transfer film, parallel to the sliding direction, to provide lubrication.

Work on amine-capped aniline trimer-modified *h*-BN nanosheet/epoxy nanocomposites also showed the importance of lubricating film under dry and water-lubricated conditions [121]. In addition, the authors highlighted the importance of the excellent mechanical properties and thermal conductivity of *h*-BN nanosheet. This is further supported by the work on 3-aminopropyltriethoxysilane-functionalized cubic BN (Fc-BN) and functionalized *h*-BN (F*h*-BN), showing the ability to reduce the expansion of cracks in epoxy-based coatings under dry sliding or seawater conditions [118]. In both works, the addition of modified *h*-BN nanosheets, Fc-BN and F*h*-BN into epoxy resin reduced the COF and WR of the nanocomposites, with 0.5 wt.% loading exhibiting the best tribological performance [118,121]. Agglomeration of BN nanofillers at high content introduced more defects for stress and thermal concentration, resulting in lower hardness and strength.

Both works demonstrated that COF and WR under water [121] and seawater [118] conditions were significantly lower than those under dry sliding. Lubrication leads to these reductions in three ways: depression on the immediate contact between the tribo-pair to reduce the adhesive wear, removal of frictional heat during the sliding to alleviate thermal softening and chemical degradation and wear debris removal to prevent abrasive wear. A comparison between Fc-BN and F*h*-BN found that Fc-BN contributes to better wear resistance, while F*h*-BN endows a low COF, regardless of the presence of lubrication. This is ascribed to the higher load-bearing ability of Fc-BN to result in harder properties of the coating, as well as better lubricity of the laminate structure of the F*h*-BN nanosheet. Owing to its hardness, a very small amount of boron carbide (B_4_C) nanoparticle, as one of the hardest ceramic materials known, just falling behind diamond and cubic BN, was also used to enhance the wear resistance of PAEK nanocomposites [45].

Transitional metal chalcogenides (TMDs), with MoS_2_ and tungsten disulfide (WS_2_) as the most popular members, emerged as a new family of 2D nanosheets. The lamellar structure of MoS_2_ consists of a sheet of Mo atoms covalently sandwiched between two hexagonally packed S layers [88,122]. Similar to graphene, a weak van der Waals force exists between layers, contributing to its superior lubricating property. These TMDs can be developed into different sizes and morphologies, such as nanosheets, nanotubes, fullerene and nanoflowers [64]. The lubricating property of MoS_2_ nanosheets drastically reduced the COF and WR of the thermoplastic PI nanocomposites [123]. Enhanced tribological properties of thermoset epoxy resin [62,122] and BMI resin [88] were also reported. COF and WR of MoS_2_/epoxy showed decrements of 78% and 76% respectively, when compared to pure epoxy [122]. Furrows, which were obviously observed on the worn surface of pure epoxy, can hardly be spotted on the surface of MoS_2_/epoxy nanocomposites [62]. However, in comparison to ZrO_2_, MoS_2_ exhibited an inferior effect on wear resistance [88]. MoS_2_-MA/PI nanocomposites were found inferior compared to CNTN/PI nanocomposites [46]. However, they are more capable in reducing the fluctuation range of COF values throughout the wear test.

Other than nanosheets, MoS_2_ nanoflowers/PI nanocomposites showed better tribological properties than pure PI under dry sliding, water- and oil-lubricated conditions [64]. With the increasing MoS_2_ content, COF of the nanocomposite coatings fluctuated slightly, while the WR decreased, reaching the minimum at 0.5 wt.% (dry and oil-lubricated sliding) and 1.0 wt.% (water-lubricated sliding), then increased. Incorporation of MoS_2_ nanoparticles also reduced COF and WR of the epoxy/PVDF blend [115]. A 10 wt.% inorganic fullerene (IF)-type WS_2_ nanoparticle is incorporated into polyvinyl alcohol (PVA) to reduce the COF value by about 70% [124]. This is attributed to the existence of out-of-plane van der Waals bonding between the different WS_2_ planes, which contributed to the low friction property. Zinc sulfide (ZnS), which is classified as metal chalcogenides, contributed to a great reduction in COF and WR of the epoxy nanocomposite coating [87]. However, ZnS nanoparticles showed a higher tendency to form aggregates in epoxy resin, thus depicting poorer COF and WR than RGO.

g-C_3_N_4_, with a graphite-like layered structure, possesses superior self-lubricating properties. It was used as a filler to effectively lower the COF and WR of PPESK film [58] and bulk PI [125]. A weak van der Waals force between sheets eased the interlaminar shearing, and also a good interaction bond between g-C_3_N_4_ and PPESK hindered the peel off of materials [58]. g-C_3_N_4_ nanosheet significantly reduced COF of PPESK compared to CNT, but not for WR.

COF of g-C_3_N_4_/PEEK nanocomposites’ variations under different lubrication regimes were investigated [126]. The lubrication regime changed from boundary, mixed to hydrodynamic lubrication as the sliding velocity increased, and COF values gradually decreased. COF of pure PEEK and its nanocomposites were nearly identical at the highest speed (0.8 m/s) because COF is governed by the oil film in the hydrodynamic lubrication regime. Whereas, under boundary and mixed lubrication conditions, PEEK has higher COF than its nanocomposites, which is ascribed to the better wettability of the lubricant, alleviating the squeezing out of lubricants from the interface. PEEK reinforced with 1 vol% g-C_3_N_4_ performed the best under the mixed lubrication condition, while higher values were observed at higher content. In particular, the tribological performance of g-C_3_N_4_/PEEK nanocomposites are comparable to that of 20-fold volume fractions of CF or micro-sized g-C_3_N_4_ particles. This is because g-C_3_N_4_ nanosheets can be easily transferred to form a more uniform and robust transfer film.

**Figure 12:**
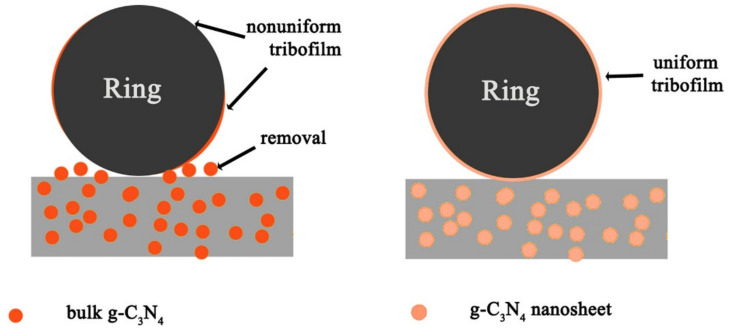
Wear mechanism experienced by g-C_3_N_4_-reinfroced phenolic coating (Reproduced with permission from L. Wu, Z. Zhang, M. Yang, J. Yuan, P. Li, F. Guo, X. Men, Tribology International; published by Elsevier, 2019) [127].

Bulk g-C_3_N_4_, nanosheet g-C_3_N_4_ and graphite of similar optimum content (1 wt.%) were filled into a phenolic coating to demonstrate different tribological performances [127]. The results revealed that g-C_3_N_4_ nanosheet/phenolic coating presented better tribological performance than bulk g-C_3_N_4_/phenolic coating, but exhibited inferior friction-reducing ability compared to graphite. This is due to the existence of interlayer hydrogen bonds in either bulk g-C_3_N_4_ or nanosheets, rather than van der Waals force only in graphite. As shown in Figure 12, the superior performance of nanosheets over bulk g-C_3_N_4_ is attributed to the smaller surface area of bulk g-C_3_N_4_ which led to weaker physical and chemical interactions with the phenolic matrix, easy removal of bulk g-C_3_N_4_ and the formation of a nonuniform transfer film. However, the wear resistance of graphite/phenolic coating was weaker than g-C_3_N_4_ as it experienced violent three-body wear combined with serious adhesive wear, evinced by a coarse worn surface with large debris.

Silicon nitride (Si_3_N_4_) is a promising ceramic material in polymer composites. Si_3_N_4_/PEEK nanocomposite coatings were fabricated on a titanium alloy substrate by electrophoretic deposition, followed by heat treatment [128]. Different cooling rates of the coatings resulted in an amorphous polymer structure or a polymer structure consisting of a combination of amorphous and crystalline structures. Coatings with a mixture of amorphous and crystalline structures have better wear resistance and lower COF than amorphous ones. Additionally, the amorphous coating had less stable cooperation with the counter-face. The greater wear intensity of the amorphous coating was supported by the presence of small grooves in the friction track and additional plastic deformation. For both structures, insignificant changes in COF were observed after the incorporation of Si_3_N_4_. The WR of amorphous Si_3_N_4_/PEEK and PEEK coatings were comparable. It is worth highlighting that the addition of Si_3_N_4_ in a coating with a mixture of amorphous and crystalline structures significantly reduced the WR by ~46% when compared with the amorphous coating.

Manufacturers have utilized silicon carbide (SiC) in high-temperature devices such as car brakes, heating machinery components and bearings. Owing to the great hardness of SiC nanoparticles, thermoplastic polymers, UHMWPE and PA6, exhibited improved wear resistance [129,130]. It was found that the incorporation of SiC into the PA6 matrix resulted in a 61% reduction of COF [130]. An ANOVA on SiC/UHMWPE nanocomposites found that loading of SiC nanoparticles is the most significant factor influencing the tribological performance of nanocomposites among other nanocomposite processing parameters [129]. The increasing percentage loading of SiC reduced the WR but increased the COF. A contradicting result was reported for SiC/OCNE thermoset nanocomposites [112]. Different filler loadings and testing conditions were carried out to investigate the effect of SiC incorporated into the OCNE matrix. The COF of the nanocomposite increased as the applied load increased, but it decreased as the nano-SiC content, sliding velocity and sliding distance increased, whereas WR increased when filler content, applied load and sliding distance increased, but decreased with increased sliding velocity. The contradicting result may be due to the different interactions between SiC and UHMWPE or OCNE.

Titanium nitride (TiN) nanopowder acted as an effective solid lubricant for HDPE nanocomposites during the wear process, reducing COF by about 12% [83]. In a later study, VTMS-treated TiN nanopowder was incorporated together with a trace amount of organic peroxide to result in a 48% reduction in WR [84]. 2D transition metal carbides with the molecular formula of Ti_3_C_2_T_x_, where T_x_ denotes the surface functional groups, was incorporated into the epoxy matrix. Incorporation of Ti_3_C_2_T_x_ nanosheets facilitated the formation of transfer film and thus improved the tribological performance of epoxy nanocomposites under the lubricated condition [109].

Some other nanofillers which are less popular, such as a triple system of bio-ceramic, CaTiZrO_5_ nanoparticles [131], nano nano-sized hydroxyapatite (nHA) [132,133], CaCO_3_ nanoparticles [134] and a complex metal alloy, Al_65_Cu_22_Fe_13_ quasicrystal (QC) [135], were incorporated into polymers to improve tribological properties. Interestingly, QC loading of 0.1%, 5% and 10% failed to improve the wear resistance of linear low-density polyethylene (LLDPE) under 147 N applied load. Only 1% QC nanoparticles apparently raised the melting point of the nanocomposite, increasing the thermal and wear resistance of the sample. Additionally, due to the formation of a protective antifriction film on the friction surface, 1% QC/LLDPE nanocomposites exhibited a stable COF value when the load was increased to 147 N. It is worth highlighting that a finite element model of a friction test on nHA/PMMA nanocomposites evinced the direct influence of load-bearing capacity of nanocomposites to its tribological performances [133]. Improved load-bearing capacity with the incorporation of nHA has induced smaller shear stress on the sample surfaces and frictional stress between sliding surfaces.

### 2.5. Hybrid Nanofillers

Hybrid polymer nanocomposites with enhanced tribological performance are produced by incorporating two or more types of nanofillers into the polymeric materials that are able to afford a synergic effect [45,53,109,132,136,137]. It is a new emerging approach to take advantage of the individual properties of each of the nanofillers. In most of the studies reported, one of the filler components is found to be a carbon-based filler. This demonstrates the increasing research trend on carbon-based fillers. Secondary hybrid nanocomposite is the most studied type of hybrid nanocomposite in this research area. However, there are also some studies which documented ternary hybrid nanocomposites.

There are several studies which documented the incorporation of two different carbon-based nanofillers to form hybrid polymer nanocomposites [39,59,138]. These secondary hybrid nanocomposites have superior tribological properties as compared to their single filler-filled nanocomposites. A study reported on graphene/short CF (SCF)/PTFE/PEEK hybrid nanocomposites found that graphene does not only enhance the load-bearing capacity, but also promotes the formation of a uniform transfer film with great strength [139]. At the same concentration, it contributed to better tribological performance than graphite. MWCNT and graphite nanopowder were incorporated together with CF to offer superior components’ bonding in ternary hybrid epoxy nanocomposites [140]. A coiled and more stable structure enhanced the heat adsorption, strength and stiffness of the nanocomposite, fabricating a high wear-resistant material. Their lubricating effect also alleviates the adhesion of nanocomposites on the counter-face, resulting in steady COF.

The effects of hybrid fillers on tribological performance of nanocomposites are investigated under different sliding temperatures, speed and load. COF was reduced but WR increased with increased sliding temperature (25–150 °C) under 4 MPa and 2 m/s [139]. At high temperature, the polymer matrix softens, and the shear force decreases, lowering COF but making material removal easier. Due to the higher thermal conductivity of composites, graphene contributed more wear resistance enhancement at high temperatures. PEEK chains around graphene began to vibrate and straighten out at high temperature, causing increments of the mean free path and phonon propagation length, which consequently elevate the thermal conductivity. Under 1 m/s and 25 °C, the COF value of hybrid nanocomposites slightly decreased as the applied pressure was increased from 1 to 4 MPa, in contrast to the gradually increasing WR trend. Higher applied pressure tends to generate more friction heat, leading to easier shear-off of the nanocomposites and promoting the formation of the transfer film. A decreasing trend of COF and WR with increasing sliding speed (1–2 m/s) was observed under 4 MPa and 25 °C. Transfer film formation is promoted at high sliding speed, while the number of the adhesive points was reduced, causing less adhesive force.

Besides, several works have been documented on hybridizing carbon-based nanofillers with nanoclay. The study on MMT/graphene/epoxy nanocomposites found that the incorporation of MMT exfoliated graphene to further improve the interfacial interaction [72]. Incorporation of 1.5 wt.% of CNT and C15A each into the UHMWPE matrix bridged to hold the polymer chains together and increase the load-bearing capacity of the nanocomposite [141]. Addition of hybrid filler reduced WR under dry and water-lubricated conditions, exhibiting smooth wear tracks with negligible ploughing. Under water lubrication, the platelet structure of nanoclay formed a torturous path to the diffusion of water molecules, alleviating the softening of the polymer. Instead of a bulk nanocomposite, the same combinations of hybrid nanocomposites were studied in coating form [55]. Since nanocomposite coating has greater hardness, it has 24% higher COF. This secondary hybrid UHMWPE nanocomposite coating exhibited a longer wear life of ~100,000 cycles under normal load of 12 N. However, higher PV factor and higher CNT content both deteriorated the wear life as it agglomerated and led to a two-phase structure in the coating.

Hybrid oxide nanoparticles with carbon-based filler were reported for PEEK-, PTFE- and epoxy-based nanocomposites. Preparation was carried out using nanosized bismuth (III) oxide (Bi_2_O_3_), CuO, SiO_2_, ZrO_2_, SiC and WS_2_ to be incorporated into PEEK/SCF composites [142,143]. The addition of hybrid CuO/SiO_2_ and Bi_2_O_3_/SiO_2_ nanoparticles into SCF/PEEK composites reduced the WR and COF by forming a transfer film on the roughness grooves and plateau areas of the counter-face. The synergic effect between the soft nanoparticles (Bi_2_O_3_ and CuO) and hard nanoparticles (SiO_2_) led to the formation of a uniform and compact transfer film that exhibited a slippery feature (easy-to-shear) and enhanced load-bearing capability during the wear process. Grafting of nano-SiO_2_ on SCF also promoted high-quality transfer film formation by improving the interfacial bonding strength between SCF and the PEEK matrix [144].

On the other hand, hybrid WS_2_/SiC nanoparticles provide better wear resistance to PEEK/SCF nanocomposites, due to the formation of a thinner transfer film with a more enhanced “easy-to-shear” characteristic, as compared to the former hybrid nanoparticles. It is reported that SiC nanoparticles transformed into SiO_2_, resulting from tribo-oxidation, thus yielding similar effect as SiO_2_ during the steady friction stage. SiO_2_ increased the load-bearing capability of the transfer film, which acts as the protective layer in limiting the direct contact between the PEEK nanocomposites and the counter-face. Instead of dry sliding, β-SiC/SCF/PEEK nanocomposites undergo sliding under lubricated conditions with simulated body fluid (SBF) [145]. The formed transfer film was still able to fill and cover up the grooves and plateau areas of the counter-face. Other than tribo-products of β-SiC and carbon species of SCF, corrosion products from the steel and calcium phosphate precipitated from SBF also played a role in performance enhancement.

In the study of Wu et al., CuO nanosheets were successfully synthesized using GO as a template [116]. The synthetic CuO nanosheets, commercial CuO nanogranules and CuO nanorods were individually incorporated into CF-reinforced PTFE composites. Addition of CuO nanogranules increased the WR, but CuO nanorods slightly reduced it. Generally, the increasing content of both fillers increased COF, WR and contact temperature. CuO nanosheets greatly reduced the WR of the hybrid nanocomposites but contributed to a lesser effect on COF. Although the incorporation of CuO nanosheets increased the contact temperature when compared to CF/PTFE composites, it enhanced the heat capacity property and showed the lowest temperature among the nanofillers. Incorporation of CuO nanosheets turned rough transfer film into smooth and compact film by enhancing the interfacial strength between CF and the PTFE matrix. It also increased the bonding strength between the film and the counter-face.

Graphene was functionalized and titanium dioxide (TiO_2_) was hydroxylated before fabricating hybrid graphene/TiO_2_/PVDF nanocomposites in order to improve the interfacial interaction between fillers and the matrix [146]. At optimized content of both fillers, the best surface roughness, transfer film thickness and transfer film adhesion on the sliding counter-face were observed. Besides functionalizing fillers, growth of nano-manganese oxide (MnO_2_) on CF has been reported to be effective in enhancing the binding force between CF and nitrile rubber-modified phenolic resin [147]. The strong interfacial bond strength improved the friction stability and wear resistance but increased the COF regardless of different applied load.

Different fabrication methods used in producing polymer nanocomposites is another factor in affecting tribological performance. Two different hybrid fillers of the same component: molybdenum trioxide (MoO_3_) nanobelts/GO and MoO_3_/GO films (*f*-MoO_3_/GO), were incorporated into a glass fiber (GF)/epoxy composite [148]. MoO_3_/GO/GF/epoxy nanocomposites were fabricated through the vacuum resin transfer mold (VARTM) method, which successfully reduced WR and COF of GF/epoxy composites. However, numerous microcracks appeared on the worn surface and the hybrid nanocomposites failed to form a continuous transfer film on the counter-face. Thus, before VARTM fabrication, MoO_3_/GO were coated on a porous PVDF membrane to form *f*-MoO_3_/GO with better thermal conductivity and hardness. A smoother and more uniform transfer film on the counter-face and negligible microcracks on the worn surface were observed after the sliding of hybrid *f*-MoO_3_/GO/GF/epoxy nanocomposites.

Another work on surface-modified CNTN/MoS_2_-MA nanohybrid/PI compared the tribological properties of physically (CNT-MoS_2_) and chemically (CMS) hybridized nanofillers [46]. CMS/PI demonstrated the lowest COF and WR among all the samples, while CNT-MoS_2_/PI exhibited inferior tribological performance compared to CNTN/PI but superior compared to virgin PI and MoS_2_-MA/PI. This evinced that chemical combination of the filler contributed to better interfacial compatibility between fillers and the matrix, and thus maximized the transfer assistance effect of CNT. CMS promoted the formation of smooth and integrated transfer film, while the transfer film of CNT-MoS_2_/PI is less homogenous. The CMS/PI specimen underwent fatigue wear, while CNT-MoS_2_/PI showed intense adhesive wear.

Hybrid carbon-based filler/MoS_2_ can commonly be synthesized through hydrothermal reaction. Its application is mostly reported in thermoset polymers, such as epoxy, thermoset PI and BMI resin. The only carbon-based filler/MoS_2_-filled thermoplastic PI was documented in the work of Chen et al. [123]. Hybrid CF/MoS_2_/PI nanocomposites are more thermally stable and resistant to frictional heat compared to their counterpart. The hybridization further decreased the WR but showed no great influence on COF as compared to MoS_2_/PI, while it reduced both values when compared to CF/PI. This suggests that the reinforcing effect of CF reduced the formation of wide grooves and debris on the worn surface, while the lubricating effect of MoS_2_ formed a smooth worn surface covered with MoS_2_ film. Comparable studies used CNF instead of CF for epoxy resin [62,122]. MoS_2_ reduced the stress on CNF surface, and in turn, CNF provided strong support for MoS_2_. Additionally, MoS_2_ enhanced the surface roughness of CNF, and increased the interfacial bonding between hybrid filler and the matrix, while hybridization improved the dispersion of MoS_2_ in epoxy resin, and subsequently improved its hardness. It is worth mentioning that the CNF to MoS_2_ ratio has a great impact on the dispersion and assembly of MoS_2_ on CNF in the core-shell structure [62]. Both COF and WR initially increased with the increase in CNF to MoS_2_ ratio, before decreasing.

**Figure 13:**
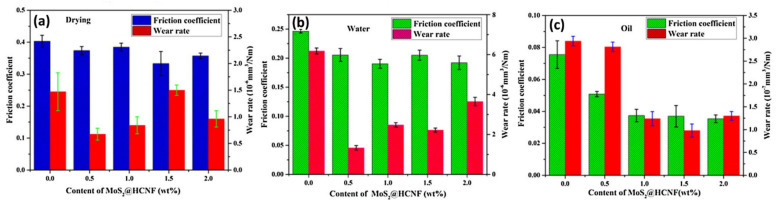
Friction coefficient and wear rate of the MoS_2_/HCNF/PI coating under (**a**) dry sliding, (**b**) water lubrication and (**c**) oil lubrication (Reproduced with permission from H. Yuan, S. Yang, X. Liu, Z. Wang, L. Ma, K. Hou, Z. Yang, J. Wang, Composites: Part A; published by Elsevier, 2017) [64].

MoS_2_/HCNF hybrid filler was incorporated into a PI coating through in situ polymerization [64]. Figure 13 illustrates the tribological properties of the MoS_2_/HCNF/PI coating at different sliding conditions. Generally, PI and its hybrid nanocomposites exhibited the lowest WR under dry sliding, followed by oil- and water-lubricated conditions. As discussed previously, diffusion of lubricants into the PI matrix has deteriorated its mechanical properties, causing more materials to be pulled off. The lowest COF values were observed under oil lubrication, followed by water lubrication then dry sliding. This indicates that oil lubrication has the most pronounced cooling and lubricating effects.

Secondary and ternary hybrid nanofillers, namely CNT/GO, CNT/MoS_2_ and CNT/GO/MoS_2_, were incorporated into epoxy resin to compare their friction and wear enhancement [59]. Ternary hybrid nanofillers have the lowest COF and WR among all samples. Similar results were observed for secondary RGO/MoS_2_ and ternary NH_2_-RGO/MoS_2_/ZrO_2_ hybrid BMI nanocomposites [88]. It is noted that the reduction in COF is attributed to ZrO_2_, which acted as a spacer to exfoliate RGO and MoS_2_ nanosheets, while the bearing property of ZrO_2_ improved the wear resistance. The tribological properties significantly improved, reaching a minimum at 0.4 wt.% of nanoparticles, before deteriorating. The wear mechanism transformed from the combination of adhesive and fatigue into abrasive wear after the incorporation of the ternary hybrid filler. CNT/GO/MoS_2_/epoxy nanocomposites were also tested on different applied loads and sliding speeds [59]. COF and WR both increased at the higher PV factor. Similar results were observed for CF/MoS_2_/PI [123]. When a heavier load was applied, the worn surface displayed furrows and long cracks because stress concentrated on the crack tip caused crack propagation. High sliding speeds caused softening of materials and increased the contact area of the tribo-pair, consequently causing more serious adhesive wear and holes on the worn surface.

Hybrid carbon-based nanofiller/*h*-BN is another popular hybrid filler for thermoplastic nanocomposites [120]. Tribological behaviors of 5 vol% *h*-BN/10 vol% SCF/PEEK hybrid nanocomposites were explored under deionized water and seawater lubricated conditions [149]. Specimens exhibited better wear resistance in seawater than in deionized water with similar COF values. This is attributed to the formation of CaCO_3_ and Mg(OH)_2_ on the rubbing interface under the seawater condition. Comparing with SCF/PEEK, a more continuous and robust boundary film was observed, covering the counter-face. The film comprised of crystalline and amorphous structures, containing three layers: amorphous carbon derived from the PEEK matrix, B_2_O_3_ and CaCO_3_ derived from hydrolysis reactions of *h*-BN nanoparticles and the reaction of seawater with the interface, and lastly, iron (III) oxide (Fe_2_O_3_) and chromium (III) oxide (Cr_2_O_3_) crystals manifested corrosion products of the steel counter-face. B_2_O_3_, which was present in significant fraction, was found responsible for enhancing the load-bearing capability of the boundary film.

**Figure 14:**
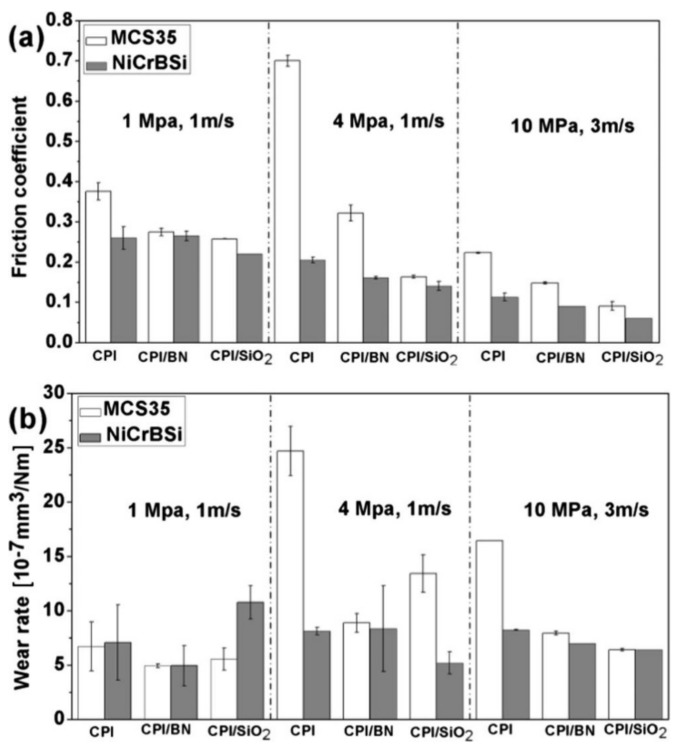
(**a**) Average friction coefficient and (**b**) wear rate of CPI and CPI hybrid nanocomposites when sliding against MCS35 and NiCrBSi at a PV factor of 1, 4 and 30 MPa·m/s (Reproduced with permission from H. Qi, G. Li, G. Liu, C. Zhang, G. Zhang, T. Wang, Q. Wang, Journal of Colloid and Interface Science; published by Elsevier, 2017) [150].

Studies on SiO_2_/*h*-BN/conventional thermoplastic polyimide (CPI) highlighted the effects of different tribo-pairs on tribological performance [150]. CPI used in the study is readily incorporated with 10 vol% of polyacrylonitrile-based SCF and 8 vol% graphite flakes. Two different counter-faces, medium carbon steel (MCS35) and alloy nickel chrome boron silicium (NiCrBSi) coating, were used. In most cases, CPI and its hybrid nanocomposites exhibited higher performance when slid against the NiCrBSi coating. However, Figure 14 shows that the COF and WR reduction effects of nanofillers are greater when slid against MCS35. The incorporation of hybrid nanofillers reduced the influence of the counter-face material. During the wear process, release of nanoparticles onto the contact surface can mitigate tribo-oxidation of metallic counter-faces by abrading the tribo-oxidation layer, which can lead to the increase of COF. The released nanoparticles are mixed with polymer particles and tribo-oxidation products to form a transfer film on the counter-face. In comparison with CPI/SiO_2_ tribo-film, CPI/*h*-BN tribo-film is less resilient and covers lesser area on the counter-face. A more severe tribo-oxidation occurred in the sliding with CPI/*h*-BN since *h*-BN is less abrasive than SiO_2_ to remove the tribo-oxidation layer. SiO_2_ nanoparticles are more readily tribo-sintered into compact layers than *h*-BN, attributed to the lower melting temperature, abundant hydroxyls and residual unsaturated bonds on the surface.

Hybrid carbon-based nanofiller/ZnS, hybrid RGO/ZnS [87] and CNT/ZnS [60] exhibited better friction-reducing and anti-wear properties than their single nanofiller-filled epoxy nanocomposites. Hybrid nanocomposites showed the narrowest wear track with the smoothest worn surface. In the case of hybrid RGO/ZnS, RGO nanosheets provided support to ZnS and improved the dispersibility and stability in epoxy resin [87]. Additionally, the rolling and sliding phenomena along the direction of the shear force is under the control of RGO nanosheets. For hybrid CNT/ZnS, CNT acted as a “micro-roller” to reduce friction, whereas ZnS loaded some stresses applied on CNT and lubricated the contact surface [60].

Its COF and WR variation with filler content is consistent with the variation of CNT/epoxy and acid-CNT/epoxy. At heavier applied load, higher COF and WR were observed due to the plastic deformation that promotes adhesive wear and penetration of the friction pair. When applied load exceeded 1.5 N, chemical degradation and thermal ageing occurred, which rapidly increased WR. Nonetheless, increasing the sliding rate lowered the COF, but raised WR. In contrast to CNT/ZnS/epoxy nanocomposites, COF of C_3_N_4_/CNT/PPESK nanocomposites film gradually decreased, and WR increased with the increase in applied load [58]. Similar results were observed in graphene/basalt fabrics/epoxy nanocomposites [151]. At higher sliding speed, the hybrid nanocomposite film demonstrated higher COF and WR values. Even so, the tribological properties of g-C_3_N_4_/CNT/PPESK film under high PV were superior compared to those of pure PPESK at low PV.

Nanoclay is often hybridized with fillers of different structures and morphologies to improve tribological properties [152,153]. Increasing MMT content in MMT/silk fibers (SF)/HDPE hybrid nanocomposites reduced the formation of wear debris and increased the protection over SF to afford lower COF and WR [152]. When applied load increased from 10 to 30 N, COF decreased, but WR increased. The debris formation and breakage of SF were promoted at higher applied load. It was found that the tribological performances of epoxy hybrid nanocomposites filled with MMT intercalated by hydroxyl-terminated polybutadiene-based quaternary ammonium salt (HTPB/QAS/MMT) were vulnerable to different curing systems [154]. COF variation of epoxy nanocomposites cured with 2-ethyl 4-methyl imidazole (2E4MI) is more sensitive to the filler content than that cured with 4,4′-diamino diphenyl methane (DDM). In the DDM-cured system, at lower content (3–6 phr), HTPB/QAS/MMT/epoxy nanocomposites showed higher COF value than pure epoxy as the roughness increased after the incorporation of filler. However, at higher fractions (18–30 phr), HTPB/QAS/MMT improved the load-carrying ability of the nanocomposites and lowered the COF below pure epoxy. The trend of WR as a function of filler content initially increased until 6 phr, and then remained stable. However, COF and WR of the 2E4MI-cured system drastically decreased with the increasing filler content. These different results are ascribed to the changes of the actual network structure and viscoelasticity caused by 2E4MI.

## 3. Summary of Tribological Performance of Polymer Nanocomposites

**Figure 15:**
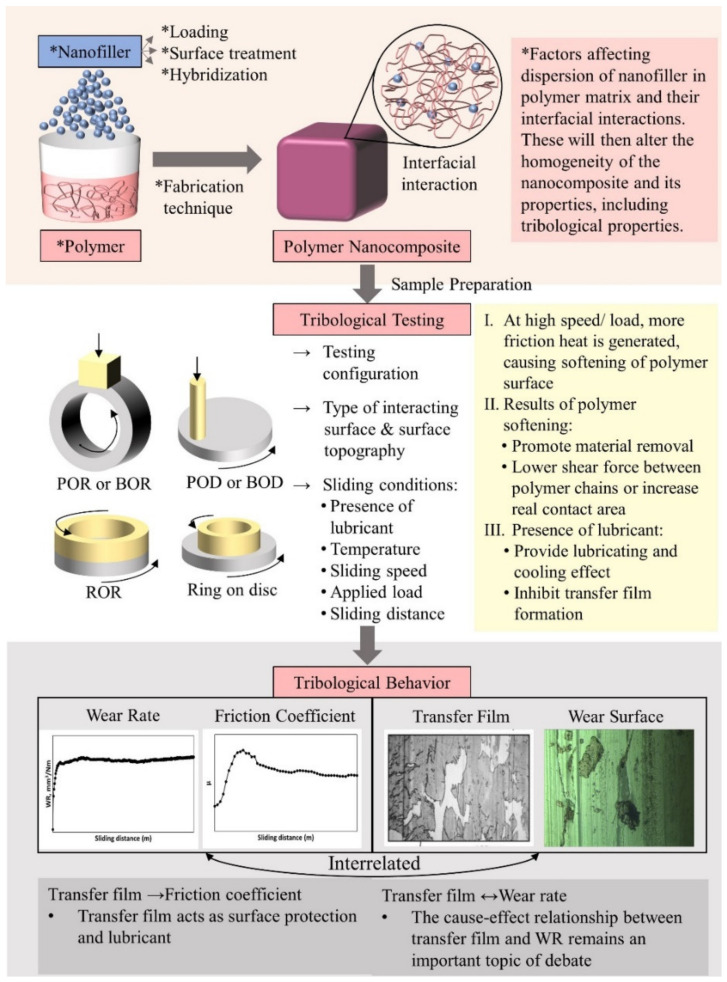
A basic tribosystem for polymer nanocomposites.

Recent research on the tribological performance of polymer nanocomposites is summarized in Table 1. The most popular polymers in recent research on tribology are PTFE, PEEK and UHMWPE. PTFE- and PEEK-based nanocomposites materials exhibited high potential for high-load-bearing systems operating in extreme conditions, where lubricants failed, for example in oil and gas industry, air-conditioning and refrigeration industry [10,21]. Due to their excellent biocompatibility, and mechanical and tribological properties, UHMWPE nanocomposites have high potential in total hip arthroplasty and joint implants [155]. In comparison with other polymers, PTFE and HDPE are capable of forming thin and highly oriented transfer film against a smooth metal surface [20]. From Table 1, it can be observed that most of the nanomaterials contributed to favorable tribo-performance as compared to their pristine resins by enhancing their mechanical properties and thermal conductivity, limiting adhesive wear with the counter-face and reducing wear debris size. Optimum composition of nanofiller is very crucial. Very low loading can yield an insignificant effect, whereas adverse effects may be observed at high content. However, in some nanomaterial-polymer systems, such as Al_2_O_3_/HDPE and SiO_2_/epoxy, nanofillers do not act as solid lubricants and showed high COF due to the increased shear force at the sliding regions of real contact, whilst an unsuitable fabrication technique can result in high-porosity nanocomposites with inferior mechanical properties, and high WR. Additionally, the exposure of nanofillers of favorable size and interfacial interactions with the matrix on the sliding surfaces can increase the robustness and lubricating effect of the transfer film, as well as inducing better adhesion between the transfer film and the counter-face. However, some works reported retarded transfer film formation due to the improved wear resistance by nanofillers. As shown in Figure 15, tribological behaviors are interrelated with wear mechanism and transfer film formation. Most works suggested that transfer film formation alters the contact dynamic of the tribo-pairs from plowing of asperities into sliding of two smooth polymeric surfaces, thus reducing wear. Nevertheless, some found that WR is independent of the condition of the transfer film upon which it is slid, and a non-continuous transfer film is the consequence of low WR [102,153]. Figure 15 also shows the effect of sliding conditions on tribological behaviors of a polymer nanocomposite. A direct comparison on the friction- and wear-reducing ability of different types of nanofillers is not feasible as their performance is versatile regarding the nature of the matrix, and their interfacial interaction with the matrix and sliding conditions. The tribological research in these fields is still at a relatively early stage.

## 4. Challenges and Future Developments

According to the reviewed studies, a few points are highlighted below to emphasize the challenges faced during the developments of polymer nanocomposites for tribological applications, which might then expand new research opportunities in the future.

- Increasing nanofiller content contributed to a positive effect on the tribological performance until agglomeration occurs. Not only that, nanofillers hybridization of different structures can improve dispersibility of nanofillers in the matrix, and different fabrication techniques also influence the dispersibility. Hence, more tribological studies should be conducted on the effects of the fabrication techniques of polymer nanocomposites, either in bulk, film or coating form.
- Owing to the viscoelasticity of polymer, high operating temperature and friction heat generation often limit the tribological applications of polymer nanocomposites. However, there is a lack of research on the contact temperature between tribo-pairs, the thermal conductivity and the stability of polymer nanocomposites at extreme operating conditions (applied load, sliding speed, temperature). Thus, more work in this field is suggested to widen the applications of polymer nanocomposites.
- Only few works have reported that the tribo-chemical reaction occurs during the sliding process. Different operating conditions and combinations of different tribo-pairs can result in distinct reactions. These reactions have a significant impact on tribological performance, and thus should be further explored.
- With the increasing concern in environmental sustainability, future works can focus on the tribological performance of polymer nanocomposites reinforced with greener nanofillers, such as natural nanofibers and wollastonite nanofibers derived from wastes.
- As tribological properties of polymer nanocomposites are not innate, the advancement of polymer nanocomposites for tribological applications is limited by the lack of a model that is capable of relating mechanical, thermal and tribological properties of polymer nanocomposites. Such model would be very useful in supporting product development with lower cost and time.

## Figures and Tables

**Table 1:** Tribological properties of polymer nanocomposites based on types of nanomaterial.

Polymer	Nanofiller	Test Conditions	Wear Rate	Friction Coefficient	Reference
Carbon-Based Nanofillers					
PTFE	CNT	Dry; AL: 101 kPa	N/A	−25%	[40]
PEEK	MWCNT OD: 10–15 nm; L: 0.1–10 µm	BOD (R); AISI E52100 stainless-steel ball; Dry; AL: 10 N; SV: 5 Hz; SD: 10,000 cycles	+142%	−67%	[42]
	Graphene Thickness: 0.34–100 nm		+121%	−56%	
POM copolymer	Pure MWCNT	POD; Steel; Dry; AL: 15, 25, 35 N; SV: 1 m/s; ST: 30 min; Ra: 0.25 µm	−9%	−20%	[47]
	Acid-treated MWCNT		−19%	−19%	
	Silanized MWCNT		−45%	−27%	
	Carbonylated MWCNT		−28%	−21%	
	Aminated MWCNT		−31%	−22%	
UHMWPE	Fluorinated MWCNT L: 2 mm; OD: 8–15 nm; ID: 4–8 nm	POD; Steel; Dry; AL: 32 N; SV: 2.6 m/s; SD: 2355 m	−95%	−27%	[48]
Epoxy resin	MWCNT D: 10–50 nm	POD; 316L steel ball; Dry; AL: 5 N; SV: 2 Hz; SD: 5 mm	−83%	−31%	[49]
POM copolymer/PTFE blend	Silanized MWCNT	POD; Steel; Dry; AL: 15, 25, 35 N; SV: 1 m/s; ST: 30 min; Ra: 0.25 µm	−35%	+23%	[50]
PA11	MWCNT D: 10–12 nm	POD; Steel disk; Dry; AL: 5, 10 N; SV: 150 rpm; ST: 10 min	−11%	N/A	[51]
Epoxy resin	MWCNT L: 1–10 µm; Number of walls: 3–15	BOD; Bearing steel SAE 52,100 balls, Dry; AL: 2 and 4 N; SV: 0.28 m/s (1000 rpm)	−36%	−78%	[52]
	C_70_		−71%	−39%	
Vinyl ester resin	MWCNT L: 10–20 µm; OD: 8–15 nm; ID: 3–5 nm	POD; Steel 42CrMo4 disc; Dry; AL: 10–20 N; SV: 0.5–1.5 m/s; SD: 1600 m	+167%	−43%	[57]
	SWCNT L: 5–30 µm; OD: 1–2 nm; ID: 0.8–1.6 nm		−33%	+11%	
UHMWPE/PP-b-LLDPE blend	Taunit CNF D: 60 nm	POD; ShKh15 steel; Dry; AL: 160 N; SV: 0.3 m/s	−80%	−46%	[61]
TPU	CNF	POD; Metal; Dry; AL: 1 kg; SV: 0.5 m/s; SD: 1000 m; ST: 33.3 min	−94%	−72%	[63]
ATSP	Graphene D: 25 µm; Surface area: 120–150 m^2^/g	POD; E52100 bearing steel pin; Dry; T: 25–300 °C; AL: 135 N (4 MPa); SV: 1 m/s (530 rpm); SD: 3603 m; ST: 60 min	N/A	−52%	[66]
UHMWPE	Graphene D: 10 µm; Thickness: 180 nm	POD; Hardened tool steel pin; Dry; AL: 39.0–97.5 N; SV: 0.1–0.75 m/s; SD: 1000 m; Ra: 0.43 µm	−31%	+27%	[67]
		POD; Hardened tool steel pin; Dry; AL: 2–8 MPa; SV: 0.1–1 m/s; SD: 377 m; Ra: 0.37 µm	−52%	−12%	[68]
		Ring on Disc; AISI4140 steel; Dry and base oil lubrication; AL: 0.1–3.1 MPa; SV: 1–2 m/s; SD: 750–1000 m; Ra: 0.341 µm	−46% (Dry) −83% (Base oil)	−40% (Dry) +40% (Base oil)	[69]
PEEK	Graphene Lateral size: 40 µm; Thickness: 10 nm; Number of layers ≤ 30	BOD; Alumina ball; Deionized water lubrication; T: 37 °C; AL: 5 N; SV: 0.05 m/s; SD: 4520 m; ST: 48 h; Ra: 0.05 µm	−83%	−38%	[70]
Epoxy/poly(2-butylaniline)	Graphene	BOD (R); 316L steel ball; Dry; AL: 2 N; SV: 1 Hz; ST: 20 min	−68%	−16%	[71]
PI	FG	BOD; GCr15 steel ball; Dry and seawater lubrication; AL: 10 N; SV: 5 Hz; ST: 30 min	−51% (Dry) −40.5% (Seawater)	−10% (Dry) −12.2% (Seawater)	[75]
Epoxy	Dopamine-coating nanographite	(R), Dry; AL: 5 N; ST: 30 min	−52%	−2%	[76]
PP/PP-g-MA	GO	POD; ASIS 1040 steel; Dry; AL: 10 −40 N; SV: 0.4–1.6 m/s; Ra: 0.2–0.32 µm	−78%	−44%	[78]
PA6	GO	POD (R); Cast iron; Dry; AL: 40 N; SV: 0.1 m/s; SD: 250 m	−18%	−53%	[80]
UHMWPE	GO monolayer sheets L: 3–5 µm; Thickness: 0.7–1.2 nm	POD; High carbon cobalt chromium alloy plate; Serum solution lubrication; AL: 160N; SV: 1 Hz; ST: 4 weeks; Ra: 0.01 µm	−30%	N/A	[81]
PI	GO	BOD; Dry and seawater lubrication; AL: 5 N; SV: 0.1569 m/s; ST: 30 min	−22% (Seawater)	−28% (Seawater)	[82]
Epoxy	Amino-treated GO	BOD (R); GCr15 steel; Dry; AL: 5 N; SV: 0.1 m/s SD: 5 mm; ST: 60 min	−92%	−58%	[85]
BMI resin	RGO	POD; Steel; Dry; AL: 196 N; SV: 200 rpm	−74%	−26%	[89]
Epoxy resin	EDA-RGO	BOD (R); GCr15 steel ball; Dry; AL: 5 N; SV: 4.2 Hz; SD: 5 mm; ST: 30 min	−30%	−75%	[90]
Epoxy/PTFE blend			−33%	−80%	
UHMWPE	ND particle size: 4 to 6 nm	POD; Steel; Dry; AL: 5 N; SV: 0.3 m/s; SD: 1000 m; ST: 1 h	−14%	−25%	[91]
	MTS-modified ND Particle size: 4–6 nm		−50%	−42%	
**Silicon-based nanofillers**					
UHMWPE	C15A modified with quaternary dimethyl dihydrogenated ammonium Platelet size: 8–15 µm	BOD; Stainless-steel ball; Dry; AL: 30 N; SV: 6.82 cm/s (300 rpm); SD: 68.2 m	−41%	−38%	[93]
	Nanomer I30E clay modified with primary octadecyl ammonium ion Platelet size: 15–20 µm		−30%	−31%	
	Nanomer I28E clay modified with quaternary octadecyl ammonium Platelet size: 15–20 µm,		−29%	−31%	
UHMWPE	C15A modified with quaternary dimethyl dehydrogenated ammonium	BOD; 100Cr6 steel; Dry; AL: 30, 60, 90 N; SV: 6.82 m/s; SD: 68.2 m	−43%	−36%	[94]
PA11	HNT OD: 30–70 nm; L: 1.3 μm	POD; Hardened steel; Dry; AL: 10 N; SV: 0.3 m/s; SD: 800 m	−38%	−14%	[97]
PTFE	HNT OD: 40 nm	ROR; 45 carbon steel ring; Dry; AL: 200 N; SV: 200 rpm; ST: 60 min	−98%	+40%	[96]
	HNT-PMMA		−95%	N/A	[98]
	HNT-SDS		−96%	N/A	
	HNT-COOH		−98%	N/A	
UHMWPE	Wollastonite nanoneedle	POD; Carbon steel; Dry; AL:1.9 MPa; SV: 0.5 m/s; ST: 3 h	−84%	+5%	[100]
PTFE	SNS	BOR; Steel; Dry; AL: 200 N; SV: 200 rpm; SD: 3500 m; ST: 2 h	−97%	−15%	[102]
UHMWPE	SNS	BOR; Steel ring; Dry; AL: 200 N; SV: 200 rpm; ST: 2 h	−73%	−54%	[103]
PI	MPS	BOD (R); GCr15 steel; Dry; T: 25 −300 °C; AL: 5, 10, 15 N; SV: 0.08 m/s	−83%	−48%	[104]
Epoxy	Amorphous SiO_2_ Particle size: 10–20 nm	POD; Al_2_O_3_ ball; Dry; AL: 15 N; SV: 120 rpm; SD: 500–4000 m	+1150% (Bulk) +213% (Coating)	+61% (Bulk) +100% (Coating)	[107]
**Metal oxide nanofillers**					
UHMWPE	CuO	BOR; Steel friction ring; Dry; AL: 200 N; SV: 200 rpm; ST: 2 h	Wear scar width: −33%	−34%	[33]
PTFE	Graphene	POD; Steel; Dry; AL: 151 N; SV: 0.1 m/s; SD: 1000 m	−98%	N/A	[65]
	Alumina Particle size: 27–43 nm		−99%	N/A	
PSU	PSU-grafted 𝛾-Al_2_O_3_	POD; Dry; AL: 5 N; SV: 75 rpm; SD: 50 m	−14%	−12%	[110]
PMMA	Al_2_O_3_ Particle size: 50 nm	POD; AISI 4140 steel disk; Dry; AL: 3, 6, 9N; SV: 1.5 m/s; SD: 450 m	−94% (5 vol% as basis, compared to 20 vol%)	−62%	[111]
PA	Hexagonal ZnO	BOD; WC steel ball; Dry; AL: 5N; SD: 90.9 m	−57%	−85%	[113]
UHMWPE	ZnO nanoparticles	BOD; stainless-steel ball; Dry; AL: 20N; SV: 300 rpm; ST: 90 min	−52%	+40%	[114]
Unsaturated polyester/PMMA blend	ZnO Particle size: 72 nm	POD; Stainless-steel; Dry; AL: 20N; SV: 1.58 m/s; SD: 1582.6 m; ST: 30 min; Ra: 0.5 µm	−63%	−36%	[156]
**Miscellaneous nanofillers**					
HDPE	GO Thickness: 2–3 nm; Lateral dimensions: 6–8 μm	POD; 100Cr6 steel; Dry; AL: 10 N; SV: 0.2 m/s; SD: 16,000 m	Wear volume: −56%	+29%	[83]
	γ-Al_2_O_3_ Particle size: 20 nm		Wear volume: −95%	+33%	
	Fumed Al_2_O_3_ Surface area: 100 m^2^/g		Wear volume: −89%	+75%	
	TiN Particle size: 20 nm; L: 200–300 nm		Wear volume: +6%	−13%	
HDPE	VTMS-treated GO Thickness: 2–3 nm; Lateral dimensions: 6–8 μm	POD; 100Cr6 steel; Dry; AL: 10 N; SV: 0.2 m/s; SD: 16,000 m	−79%	+3%	[84]
	VTMS-treated HNT OD: 50–70 nm; L: 200–2000 nm		−38%	+18%	
	VTMS-treated TiN Particle size: 20 nm; L: 200–300 nm		−49%	−8%	
	VTMS-treated fumed SiO_2_ Particle size: 12–15 nm		−72%	+15%	
Epoxy/PVDF blend	La_2_O_3_ D: 50 nm	BOD (R); Carbon steel; Dry and hydraulic oil lubrication; AL: 17.6 N; SV: 0.024 m/s; SD: 6 mm; ST: 10 min for dry sliding, 20 min for lubricated condition	−91% (Dry) −59% (Oil)	−18% (Dry) −55% (Oil)	[115]
	MoS_2_ D: 50 nm		−79% (Dry) −33% (Oil)	−62% (Dry) −49.44% (Oil)	
PA−6	Cu/Si	BOR; Steel ring AISI 1045; Dry; AL: 150 N; SV: 150 rpm; ST: 1 h	Wear scar width: −41%	−26%	[117]
Epoxy resin	Fc-BN	BOD (R); Si_3_N_4_ ball; Dry and seawater lubrication; AL: 5 N; SV: 5 Hz; SD: 5 mm; ST: 20 min	−75% (Dry) −70% (Seawater)	−10% (Dry) −30% (Seawater)	[118]
	F*h*-BN		−74% (Dry) −68% (Seawater)	−12% (Dry) −39% (Seawater)	
PAEK	*h*-BN Thickness: 50 nm	POD; EN 31 steel; Dry; AL: 0.5–3.0 MPa; SV: 1 m/s; SD: 5000 m	−96%	+10%	[119]
Epoxy resin	Amine-capped aniline trimer-modified *h-*BN	POD; 316L steel ball; Dry and water lubrication; AL: 5 N; SV: 2 Hz; SD: 5 mm	−29% (Dry) −88% (Water)	−13% (Dry) −36% (Water)	[121]
PVA	IF-WS_2_ Particle size: 80–160 nm	Ball on 3 Plates; Steel; Dry; AL: 10 N; SV: 0 to 1 m/s	N/A	−70%	[124]
PI	g-C_3_N_4_	BOD (R); stainless-steel ball (GCr15); Al: 2, 4, 50 N; SV: 0.42 m/s; ST: 10 min	−19%	−11%	[125]
PEEK	g-C_3_N_4_	POR; Bearing steel ring (GCr15); PAO4 oil lubrication; AL: 400 N; SV: 0.03–0.8 m/s; ST: 3 h; Ra: 0.1–0.2 µm	−62%	−60%	[126]
Phenolic resin	g-C_3_N_4_	BOR; Steel ring; Dry; AL: 320 N; SV: 2.5 m/s; ST: 1 h	−47%	−2%	[127]
PEEK	Si_3_N_4_ Particle size: 15–30 nm	BOD; Al_2_O_3_ ball; Dry; AL: 5 N; SV: 0.05 m/s; SD: 2000 m	−16% (Amorphous as basis, compared with coating consisting of a mixture of amorphous and crystalline structures)	−70% (Amorphous as basis, compared with coating consisting of a mixture of amorphous and crystalline structures)	[128]
UHMWPE	SiC	POD; Silver steel pin; Dry; AL: 64 N; SV: 0.5 m/s; SD: 500 m; Ra: 0.43 ± 0.04 µm	−22% (1 wt.% as basis, compared to 7 wt.%)	+6% (1 wt.% as basis, compared to 7 wt.%)	[129]
PA−6	SiC Particle size: 40 nm	304 stainless-steel; Dry; AL: 5 N; SV: 180 rpm	N/A	−61%	[130]
PMMA	CaTiZrO_5_	POD; Dry; AL: 5N; ST: 5, 10, 15 min	−88%	N/A	[131]
PMMA	nHA	POD (R); Stainless-steel; Dry; AL: 3, 6, 9, 12 N; SV: 0.4 m/s; SD: 5 cm	−34%	−19%	[133]
		POD (R); PMMA disk; Dry; AL: 3, 6, 9, 12 N; SV: 0.4 m/s; SD: 5 cm	−35%	−26%	
PP	CaCO_3_ Particle size: 16nm	Steel; Dry; AL: 30 N	N/A	−30%	[134]
LLDPE	Al_65_Cu_22_Fe_13_ quasicrystals D: 0.01–3 µm	POD; Steel pin; Dry; AL: 47, 98, 147 N; SV: 25 rpm; SD: 2250 m	−57%	−58%	[135]
Hydroxypropyl methylcellulose	Aluminum nanoparticles D: 110 nm	BOD: Chrome steel ball; Dry; AL: 2 N; SV: 3mm/s, SD: 30 m	−90%	−70%	[157]
**Hybrid nanofillers**					
PTFE	MWCNT D: 8–15 nm; L: 50 mm	POD; Dry; AL: 100 MPa; SV: 0.262 m/s	−33%	−3%	[39]
	GO		−36%	−3%	
	Hybrid MWCNT/GO		−43%	−6%	
PAEK	–COOH-functionalized MWCNT OD: 20 nm; ID: 16 nm; L: 20 µm	POD; EN31 alloy steel; Dry; AL: 20, 30 N; SV: 1, 2 m/s; SD: 600, 1200 m; ST: 10 min	−57%	N/A	[45]
	B_4_C Particle size: 30–60 nm		−57%	N/A	
	Hybrid B_4_C/MWCNT–COOH		−71%	N/A	
PI	CNTN OD: 8–15 nm; L: 50 µm	BOD (R); Stainless-steel ball; Dry; AL: 6 N; SV: 10 Hz; SD: 10 mm; ST: 10 min	−76%	−26%	[46]
	MoS_2_-MA Particle size: 100 nm		−39%	−17%	
	Hybrid CNT-MoS_2_		−61%	−22%	
	Hybrid CMS		−84%	−31%	
UHMWPE	SWCNT D: 40–60 nm	BOD; Stainless-steel ball; Dry; AL: 7–15 N; SV: 0.1 m/s; SD: 3600–50,000 cycles	N/A	−54% (Compared to uncoated titanium)	[53]
	Hybrid SWCNT/HA Thickness of HA: 0.3–0.5 µm	BOD; Stainless steel ball; Dry; AL: 12 N; SV: 0.1 m/s; SD: 34,000–250,000 cycles	−88% (Compared to uncoated titanium)	−57% (Compared to uncoated titanium)	
UHMWPE	C15A modified with quaternary dimethyl dihydrogenated ammonium Platelet size: 8–15 µm	BOD; Stainless-steel ball; Dry; AL: 5–12 N; SV: 0.1–0.3 m/s; SD: 125 m	−48%	+6%	[54,55]
	Hybrid C15A/MWCNT D: 23 nm	BOD; Stainless-steel ball; Dry; AL: 5–15 N; SV: 0.1–0.3 m/s; SD: 125–1300 m	−98%	N/A	
PPESK	CNT OD: 50 nm; L: 15 µm	BOD; 440c stainless-steel ball; Dry; AL: 2, 5, 8 N; SV: 0.042, 0.083, 0.126 m/s; ST: 20 min	−76%	−38%	[58]
	g-C_3_N_4_		−47%	−42%	
	Hybrid g-C_3_N_4_/CNT		−84%	−65%	
Epoxy resin	CNT OD: ≥50 nm; L: 10–20 µm	BOD; 440c stainless-steel ball; Dry; AL: 3–6 N; SV: 200–500 rpm; ST: 20 min	−91%	−16%	[59]
	GO		−92%	−71%	
	MoS_2_		−89%	−82%	
	Hybrid CNT/GO		−94%	−80%	
	Hybrid CNT/MoS_2_		−92%	−81%	
	Hybrid CNT/GO/MoS_2_		−96%	−91%	
Epoxy resin	CNT OD: >50 nm; L: 10–20 µm	BOD; GCr15 steel ball; Dry; AL: 1.5 N; SV: 200 rpm; ST: 20 min	−86%	−10%	[60]
	Acid-treated CNT		−90%	−11%	
	Hybrid acid treated CNT/ZnS	BOD; GCr15 steel ball; Dry; AL: 0.5–2 N; SV: 200–500 rpm; ST: 20 min	−95%	−45%	
Epoxy resin	CNF	BOD; 440c ball; Dry; AL: 3–6 N; SV: 100–400 rpm; ST: 20 min	−80%	−19%	[62]
	MoS_2_		−75%	−80%	
	Hybrid CNF/MoS_2_		−92%	−90%	
PI	HCNF D: 100 nm; L: 2–20 µm	BOD; GCr15 steel (AISI 52100) ball; Dry, water and paraffin oil-lubrication; AL: 20 N; SV: 20 Hz; ST: 30 min	−30% (Dry) −56% (Water) −67% (Oil)	−10% (Dry) −27% (Water) −50% (Oil)	[64]
	MoS_2_		−69% (Dry) −61% (Water) −62% (Oil)	−11% (Dry) −24% (Water) −23% (Oil)	
	Hybrid MoS_2_/HCNF		−55% (Dry) −79% (Water) −66% (Oil)	−18% (Dry) −22% (Water) −51% (Oil)	
Epoxy	Graphene	POD; Steel; Dry; T: 25, 60, 95 °C; AL: 10 N; SV: 0.5 m/s; SD: 1000 m	−29%	−34%	[72]
	MMT		−14%	−14%	
	Hybrid graphene/MMT		−29%	−33%	
PI	GO	BOD (R); Steel ball; Dry; AL: 10 N; SV: 10 cm/s; SD: 500 m	−38%	−7%	[79]
	POSS-GO		−90%	−18%	
Epoxy	GO	BOD (R); GCr15 steel ball; Dry; AL:2, 5, 10N; SV: 0.04, 0.1, 0.4 m/s; SD: 5 mm; ST: 1 h	−47%	−11%	[86]
	Polyetheramine-functionalized GO		−94%	−54%	
Epoxy resin	RGO	BOD; GCr15 steel ball; Dry; AL: 10 N; SV: 0.033 m/s; ST: 30 min; Ra: 0.301 µm	−60%	−60%	[87]
	ZnS		−30%	−60%	
	Hybrid RGO/ZnS	BOD; GCr15 steel ball; Dry; AL: 5–25 N; SV: 0.033 m/s; ST: 30 min; Ra: 0.301 µm	−81%	−84%	
BMI resin	ZrO_2_	POR; Steel ring; Dry; AL: 196 N; SV: 200 rpm; ST: 120 min	−96%	−5%	[88]
	RGO		−80%	−17%	
	MoS_2_		−82%	−27%	
	Hybrid RGO/MoS_2_		−82%	−46%	
	Hybrid NH_2_-RGO/MoS_2_/ZrO_2_		−91%	−68%	
Epoxy	Al_2_O_3_ D: 30 nm	POR; GCr15 steel; Ultra-low-sulfur diesel lubrication; AL: 100 N; SV: 0.4 m/s; ST: 1 h	−95%	−65%	[109]
	Ti_3_C_2_T_x_		−46%	−35%	
	Hybrid Al_2_O_3_/Ti_3_C_2_T_x_		−97%	−95%	
PTFE	Hybrid CuO nanogranules/CF Particle size of CuO: 40 nm; D of CF: 20 µm; L of CF: 150 µm	ROR; AISI 1045 steel ring; Dry; AL: 250 N; SV: 1.4 m/s; ST: 2 h	−11%	+13%	[116]
	Hybrid CuO nanorods/CF D of CuO: 50 nm; L of CuO: 1.5 µm		−15%	+9%	
	Hybrid CuO nanosheets/CF Thickness of CuO: 13 nm		−51%	−6%	
POM	*h*-BN D: 100 nm	BOR; Austenitic stainless-steel; Water lubrication; AL: 50–300 N; SV: 0.445 m/s; ST: 150 min	−85%	−29%	[120]
	Hybrid *h*-BN/SCF D of SCF: 7 μm; L of SCF: 20 to 50 μm		−52% (Compared to SCF/POM)	−13% (Compared to SCF/POM)	
PI	MoS_2_	BOD (R); GCr15 alloy steel ball; Dry; AL: 3 N; SV: 0.083 m/s; ST: 30 min	−47%	−11%	[123]
	Hybrid MoS_2_/polyacrylonitrile-based CF	BOD (R); GCr15 alloy steel ball; Dry; AL: 3, 4.5 N; SV: 0.083,0.116 m/s; ST: 30 min	−63%	−10%	
PAEK/PDMS blend	nHA	POD; EN31 alloy steel; Dry; AL: 5, 30, 60 N; SV: 1.7 m/s; SD: 6000 m	−61%	+56%	[132]
	Hybrid nHA/CNF		+500%	+11%	
PA6	Hybrid GO/GF Thickness: 0.8 to 2 mm	Gear to gear; Dry; AL: 150 N; SV: 1200 rpm; ST: 8 h	−74%	N/A	[136]
Epoxy resin	Hybrid SWCNT/banana fiber	Dry; AL: 10–30 N; SV: 1–1.5 m/s; SD: 500 m	Wear loss: −63% (Compared to banana fiber/epoxy)	N/A	[137]
PA	Hybrid fullerene soot/SCF Particle size of SCF: 40–50 μm	POR (for WR); Stainless-steel; Dry; AL: 80 N; SV: 1.9 m/s 3 Ball on Plate (for COF); Stainless-steel; Dry; AL: 10–50 N; SV: 0.015–0.75 m/s; SD: 2000 mm	WR: −55.88% (Compared SCF/PA)	+14% (Compared SCF/PA)	[138]
PEEK/PTFE blend	Hybrid graphene/SCF	POD; AISI 304 stainless-steel; Dry; T: 25, 100, 150 °C; AL: 1–4 MPa; SV: 1, 1.5, 2 m/s; ST: 3 h; Ra: 0.15–0.30 µm	−39% (Compared to SCF/blend)	−54% (Compared to SCF/blend)	[139]
	Hybrid graphite/SCF		−24% (Compared to SCF/blend)	−20% (Compared to SCF/blend)	
Epoxy	Hybrid MWCNT/graphite nanopowder/SCF L of SCF: 5–10 mm	A dynamometer coupled to the Aisin Toyota 5k engine; Dry; SV: 15.5–27.8 m/s	−100%	−2%	[140]
UHMWPE	Hybrid C15A/CNT D of CNT: 25 to 26 nm	BOD; 440C stainless-steel; Dry and water lubrication; AL: 30 N (Dry), 50 N (Wet); SV: 0.06 m/s; SD: 68.2 m (Dry), 6 km (Wet); ST: 5000 cycles (Dry), 150,000 cycles (Wet)	−64% (Dry) −47% (Water)	+35% (Dry) +30% (Water)	[141]
PEEK	Hybrid CuO/SiO_2_/SCF Particle size of CuO: 30 nm; Particle size of SiO_2_: 20 nm; D of SCF: 7 μm; L/D ratio of SCF: 1:5–1:10	POR; Steel; Dry; AL: 300 N; SV: 1 m/s; ST: 5 h	−28% (Compared to PEEK/SCF)	−53% (Compared to PEEK/SCF)	[142]
	Hybrid Bi_2_O_3_/SiO_2_/SCF Particle size of Bi_2_O_3_: 80- 200 nm		−29% (Compared to PEEK/SCF)	−53% (Compared to PEEK/SCF)	
	Hybrid WS_2_/SiC/SCF Particle size of WS_2_: 20–50 nm; Particle size of SiC: 20 to 100 nm		−38% (Compared to PEEK/SCF)	−81% (Compared to PEEK/SCF)	
PEEK	Hybrid SCF-SiO_2_ D of SCF: 7 μm; L of SCF: 50 μm; Particle size of SiO_2_: 20 nm	BOR; steel ring; Dry; AL: 200, 400 N; SV: 200 rpm; ST: 2 h	−55%	−42%	[144]
	Hybrid Cenosphere/SCF-SiO_2_ Particle size of cenosphere: 2.6 μm		−87%	−56%	
PEEK	Hybrid β-SiC/SCF D of β-SiC: 35.3 ± 12.1 nm; L of SCF: 35 to 70 μm; D of SCF: 7 μm	POR; 316L stainless-steel; SBF lubrication; T: 37 °C; AL:100 N; SV: 0.1 m/s; ST: 2 h	−57%	−80%	[145]
PVDF	3-hydroxytyramine hydrobromide-functionalized graphene D: 2–3 µm; Thickness: 6–8 nm	POD; steel ball; Dry; AL: 10 N; SV: 200 rpm: SD: 12 km; ST: 1 h	−61%	−14%	[146]
	Hybrid hydroxylated TiO_2_/functionalized graphene Particle size of TiO_2_: 25 nm		−76%	−16%	
Epoxy	Hybrid MoO_3_/GO/GF	BOD; Steel; Dry; AL: 2, 4, 6, 8 N; SV: 20–120 mm/s; ST: 8 h	−59% (Compared to GF/epoxy)	−13% (Compared to GF/epoxy)	[148]
	Hybrid *f*-MoO_3_/GO/GF		−81%% (Compared to GF/epoxy)	−42% (Compared to GF/epoxy)	
PEEK	Hybrid *h*-BN/polyacrylonitrile-based SCF D of *h*-BN: 20 nm; L of SCF: 100 µm; D of SCF: 7 µm	POD (R); SUS 316 stainless-steel disc; Artificial seawater and deionized water lubrication; AL: 100 N; SV: 2 Hz; SD: 5 mm; ST: 120 min; Ra: 0.15 µm	−98% (Seawater) −98% (Deionized water)	−46% (Seawater) −51% (Deionized water)	[149]
PI	Hybrid SiO_2_/polyacrylonitrile-based SCF/graphite flake D of SiO_2_: 20 nm; L of SCF: 100 μm; D of SCF: 7 μm	POD; MCS35 or Alloy NiCrBSi coating; Dry; AL: 1, 4, 10 MPa; SV: 1, 3 m/s; ST: 5 h	−48% (MCS35) −18% (NiCrBSi)	−74% (MCS35) −27% (NiCrBSi)	[150]
	Hybrid *h*-BN/polyacrylonitrile-based SCF/graphite flake D of *h*-BN: 120 nm		−62% (MCS35) −6% (NiCrBSi)	−54% (MCS35) −9% (NiCrBSi)	
Epoxy	Hybrid graphene/basalt fiber	POD; Steel 52,100 Pin; Dry; AL: 20, 40N; SV: 0.5 m/s; SD: 1000 m	−38%	−58%	[151]
HDPE	Hybrid MMT/SF	POD; Dry; AL: 10, 20, 30N; SV: 200 rpm; SD: 3000 m; ST: 10–40 min	−23%	−33%	[152]
Epoxy	Hybrid organo-modified MMT/silane-treated E-type of plain-weave glass-woven roving fabric	POD; Alloy steel; Dry; AL: 75, 150, 300 N; SV: 1, 2, 3 m/s; SD: 2000, 6000, 10,000 m	−55% (Compared to glass/epoxy)	−45% (Compared to glass/epoxy)	[153]
Epoxy resin	Hybrid HTPB/QAS/MMT	BOR (R); AISI-C−52100 steel ring; Dry; AL: 300 N; SV: 200 Hz; ST: 20 min	−96% (2E4MI-cured) +150% (DDM-cured)	−57% (2E4MI-cured) +23% (DDM-cured)	[154]

Remark: Tribological performances were reported as compared to neat polymer or otherwise stated; D—diameter; OD—outer diameter; ID—inner diameter; L—length; POD—pin-on-disc; BOD—ball-on-disc; POR—plate-on-ring; BOR—block-on-ring; ROR—ring-on-ring; R—reciprocating mode; T—operating temperature; AL—applied load; SV—sliding velocity; SD—sliding distance; ST—sliding time; Ra—surface roughness.

## Data Availability

Not applicable.
